# Effectiveness and safety of auricular therapy for polycystic ovary syndrome: a systematic review and meta-analysis

**DOI:** 10.3389/fendo.2026.1726938

**Published:** 2026-03-04

**Authors:** Xi Li, Shuang Xu, Liangzhen Xie, Hongying Kuang, Jialing Liu, Yan Li

**Affiliations:** 1 Heilongjiang University of Chinese Medicine, Harbin, China; 2 First Affiliated Hospital, Heilongjiang University of Chinese Medicine, Harbin, China

**Keywords:** auricular therapy, auricular vagus nerve stimulation, meta-analysis, polycystic ovary syndrome, systematic review

## Abstract

**Background:**

Auricular therapy (AT) has attracted significant interest as a potential treatment for polycystic ovary syndrome (PCOS). A systematic review and a meta-analysis were conducted to evaluate the effectiveness and safety of AT in managing PCOS by analyzing evidence from randomized controlled trials (RCTs).

**Methods:**

Eight electronic databases were searched from their inception until December 22, 2024. Two independent reviewers performed study screening, data extraction, and quality assessment using the Cochrane Collaboration’s Risk of Bias tool. A random-effects meta-analysis was conducted to synthesize data from included studies using mean differences (MDs). This study was registered with the Open Science Framework (OSF) (DOI: 10.17605/OSF.IO/VBPSM).

**Results:**

This systematic review and meta-analysis, which included 18 RCTs involving 1,231 patients with PCOS, found insufficient evidence to support the efficacy of AT as a stand-alone intervention for PCOS. However, AT used as an adjunct therapy exerted beneficial effects on PCOS outcomes. For AT combined with traditional Chinese medicine (TCM) formula versus TCM formula alone, a reduction in body mass index (BMI) (MD: –0.82, 95% confidence interval (CI): –1.60 to –0.03, *P* = 0.04) was observed. Moreover, the reductions were associated with scores on the Self-rating Anxiety Scale (SAS) (MD: –3.81, 95% CI: –6.26 to –1.36, *P* = 0.002) and Self-rating Depression Scale (SDS) (MD: –4.22, 95% CI: –7.74 to –0.69, *P* = 0.02). No significant effect was identified for hormonal profiles (luteinizing hormone (LH) levels, LH/follicle-stimulating hormone (FSH) ratio, testosterone (T) levels), metabolic parameters (fasting blood glucose (FBG) levels, fasting insulin (FINS) levels, or Homeostasis Model Assessment of Insulin Resistance (HOMA-IR)), or waist-hip ratio (WHR). For AT combined with metformin versus metformin alone, a reduction was observed in BMI (MD: –0.77, 95% CI: –1.23 to –0.31, *P* = 0.0009), WHR (MD: –0.03, 95% CI: –0.05 to –0.02, *P* < 0.0001), and LH levels (MD: –0.81, 95% CI: –1.05 to –0.57, *P* < 0.0001). For AT combined with acupuncture versus acupuncture alone, a reduction was observed in BMI (MD: –3.21, 95% CI: –5.09 to –1.33, *P* = 0.0008), LH levels (MD: –0.80, 95% CI: –1.16 to –0.43, *P* < 0.0001), and HOMA-IR (MD: –0.10, 95% CI: –0.16 to –0.05, *P* < 0.0001). A reduction was also associated with the LH/FSH ratio (MD: –1.39, 95% CI: –1.76 to –1.02, *P* < 0.0001). However, no significant effect was identified for WHR, and the evidence was insufficient for the effect on FINS levels.

**Conclusion:**

Our findings suggest that adjunctive AT may be associated with improvements in key clinical outcomes, including anthropometric measures (BMI, WHR), hormonal parameters (T levels, LH levels, LH/FSH ratio), and psychological health. However, the specific benefits may vary depending on the co-intervention. Although the included studies did not report any serious adverse events, this should be interpreted with caution due to the potential for underreporting. Methodological limitations warrant careful interpretation of our findings, including a high risk of bias, high heterogeneity, and small sample sizes. These limitations highlight the need for further high-quality, well-designed, and adequately powered RCTs to confirm the efficacy and safety of AT in PCOS management.

**Systematic review registration:**

https://osf.io/vbpsm/, identifier DOI: 10.17605/OSF.IO/VBPSM.

## Introduction

The global prevalence of polycystic ovary syndrome (PCOS) among women of reproductive age reportedly ranges from 6% to 25% (1, 2), with the current prevalence in China reaching 7.8%, representing a 65% increase over the past decade (3). This rising trend highlights the growing public health burden associated with PCOS and underscores the urgent need for effective management strategies (4–6).

Current treatments for PCOS primarily focus on symptom management (7). However, the chronic nature of PCOS and the potential for adverse effects associated with these treatments highlight the ongoing need for complementary therapies that may offer improved patient well-being and long-term adherence (8–11).

In recent years, complementary and alternative therapies, such as herbal medicine (12), mind–body interventions including yoga and meditation (13, 14), acupuncture (15), and auricular therapy (AT) (16), have gained increasing research attention as approaches for managing PCOS. Notably, the 2018 and 2023 International Evidence-Based Guidelines report marked variation in care and low-to-moderate certainty of evidence for many interventions, highlighting the need for further rigorous evaluation of adjunctive, patient-centered strategies that may address metabolic risk and psychological well-being (6, 17).

AT, which involves stimulating specific points on the ear, has a long history in traditional Chinese medicine (TCM) and is now being investigated within a modern scientific framework (18). The French physician Paul Nogier first proposed the “inverted fetus” somatotopic map of the auricle in the 1950s, which laid the foundation for the standardization and global dissemination of AT (19, 20). It is now understood that the auricle is the area on the body surface that reflects the vagus nerve (VN), suggesting that AT may exert its therapeutic effects through vagal modulation, a mechanism increasingly recognized for its role in regulating metabolic and endocrine function (18, 21). Its mechanisms of action are believed to involve modulation of the autonomic nervous system and interactions with the neuroendocrine and neuroimmune systems (22–24).

Clinically, AT is commonly used as an umbrella term for interventions that stimulate auricular points, including auricular acupuncture and auricular acupressure (e.g., ear seeds or press pellets). Such modalities have been reported in PCOS-related trial protocols (25) and described in recent TCM consensus documents (26). In parallel, transcutaneous auricular vagus nerve stimulation (ta-VNS), which targets auricular regions innervated by the VN, has been explored as a neuromodulatory approach with potential relevance to the endocrine–metabolic and psychological features of PCOS, prompting further investigation into auricular-based interventions (18).

The potential benefits of AT in PCOS management are multifaceted. It has been investigated for its ability to regulate sex hormone levels (27, 28), improve insulin resistance (IR) (25), and alleviate associated symptoms such as weight gain (29) and depression (30). Beyond its therapeutic effects on these key outcomes, AT may offer distinct advantages compared to conventional treatments, such as oral contraceptives and insulin sensitizers, as well as other complementary therapies, including conventional acupuncture. These advantages include a greater safety profile (31, 32), non-invasiveness, and cost-effectiveness (33), which may contribute to improved patient compliance (34) and treatment adherence. While several studies have reported favorable outcomes of AT in treating PCOS (35, 36), the current evidence remains fragmented, and a comprehensive understanding of its efficacy and safety is lacking. Importantly, available RCTs are heterogeneous in intervention implementation and trial contexts, including differences in AT modalities, comparators, and outcome assessments (25, 37), and in some trials AT has been evaluated in combination with other interventions, such as adjuncts to lifestyle management (37) and, in some studies, as an adjunct to pharmacotherapy (38), which complicates interpretation and may contribute to inconsistent findings across outcomes.

However, existing evidence on AT for PCOS remains fragmented and heterogeneous—making it difficult to draw clear conclusions regarding its effectiveness and safety across clinically relevant outcome domains. Moreover, although a protocol for a systematic review of AT in PCOS was published in 2020 (16), an up-to-date quantitative synthesis incorporating the expanding RCT evidence base and summarizing both effectiveness and safety across clinically relevant domains remains warranted.

Therefore, the objective of this systematic review and meta-analysis was to synthesize existing evidence and clarify the effectiveness and safety of AT in treating PCOS, with analyses organized by co-intervention type and outcome domain where appropriate.

## Materials and methods

This systematic review was conducted in accordance with the Preferred Reporting Items for Systematic Reviews and Meta-Analyses (PRISMA) 2020 Statement (39). The study protocol was registered prospectively with the Open Science Framework (OSF) (DOI: 10.17605/OSF.IO/VBPSM). Ethical approval was not required for this study.

### Eligibility criteria

Studies were considered eligible for inclusion if they met the following criteria:

(a) Study design: Parallel assignment randomized controlled trials (RCTs).
(b) Population: Patients diagnosed with PCOS, provided the diagnosis met established criteria, according to those outlined by the 2003 Rotterdam Consensus Workshop of the European Society of Human Reproduction and Embryology and the American Society of Reproductive Medicine (40).
(c) Intervention: Studies investigating the use of AT for the management of PCOS, including studies where AT was used as a standalone intervention or as an adjunctive therapy combined with conventional or complementary interventions. The specific AT methods considered were ear-point pressure seeds and electrical stimulation of the auricular VN.
(d) Outcome measures: Trials that provided sufficient data for effect size estimation in the meta-analysis regarding clinical, hormonal, or metabolic outcomes.

The exclusion criteria were as follows: (a) non-randomized studies, animal studies, reviews, protocols, case reports, and conference abstracts. In the case of duplicate studies, the most comprehensive or recent version was selected; (b) women with other underlying conditions, such as congenital adrenal hyperplasia, Cushing’s syndrome, thyroid hormone abnormalities, hyperprolactinemia, ovarian/adrenal tumors, or any severe medical, neurological, or psychiatric conditions, were excluded.

### Data sources and search strategy

Two reviewers (XL and SX) independently performed a comprehensive search of the following electronic databases from their inception until December 22, 2024: PubMed, Embase, Web of Science, the Cochrane Library, China National Knowledge Infrastructure (CNKI), Wanfang Database, China Science and Technology Journal Database (VIP), and the Chinese Biomedical Literature Database (CBM). The searches were conducted without language restrictions. The search strategies were developed using a combination of keywords and controlled vocabulary (e.g., Medical Subject Headings (MeSH) in PubMed and Emtree in Embase) where available.

The detailed search strategy for PubMed was as follows:

1. “Ear acupuncture”[MeSH Terms] OR “auricular acupuncture”[MeSH Terms].
2. “ear”[tiab] OR “ear acupuncture”[tiab] OR “auricular therapy”[tiab] OR “auricular acupuncture”[tiab] OR “auricular acupressure”[tiab] OR “auricular acupoints”[tiab] OR “auricular point-sticking”[tiab] OR “auricular point pressing with bean”[tiab].
3. #1 OR #2.
4. “Polycystic ovary syndrome”[MeSH Terms] OR “Stein–Leventhal Syndrome”[MeSH Terms].
5. “Polycystic ovary syndrome”[tiab] OR “polycystic ovarian syndrome”[tiab] OR “Stein–Leventhal syndrome”[tiab] OR “polycystic ovary disease”[tiab] OR “syndrome and polycystic ovary”[tiab] OR “PCOS”[tiab].
6. #4 OR #5.
7. #3 AND #6.

The complete search strategies for each database are provided in **Supplementary Material S1**.

### Selection process

**Figure 1:**
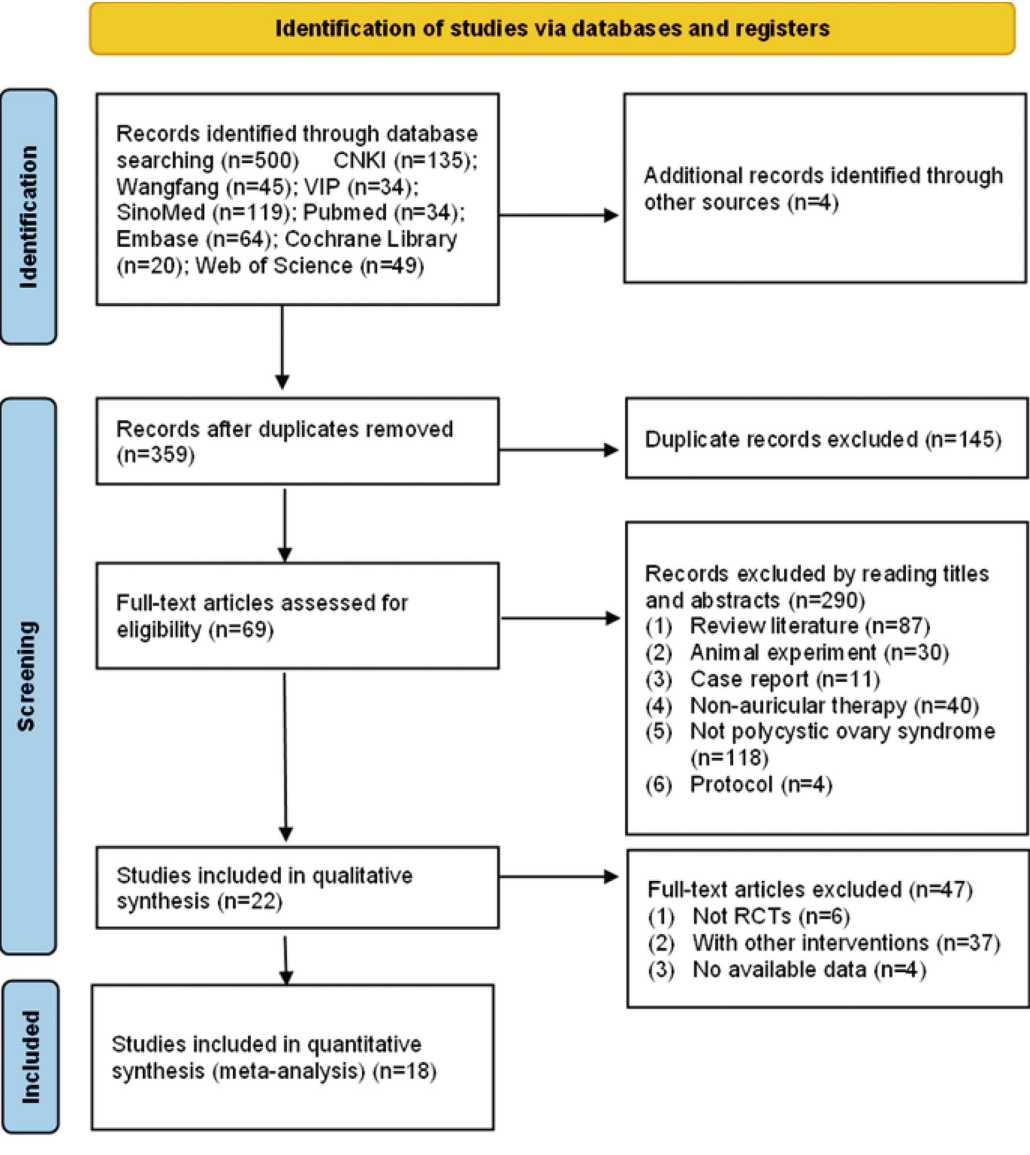
Literature screening process and results.

Two reviewers (XL and SX) independently screened titles and abstracts for eligibility and performed deduplication using EndNote 2025. Full texts of the selected studies were then reviewed, and reasons for exclusion were systematically documented. Disagreements between the two reviewers were resolved by discussion and consensus. If no consensus could be reached, a third reviewer (YL) was consulted. The study selection process is illustrated in **Figure 1**.

### Data collection

Two reviewers (XL and SX) independently extracted the following parameters from each included study: basic information (first author, publication year, and country), participant characteristics (region, sample size, mean age, and body mass index (BMI)), PCOS diagnostic criteria, AT characteristics (method, frequency, and duration), control treatment characteristics (method, frequency, and duration), clinical outcome parameters, and safety outcomes including adverse events. Any disagreements were resolved through discussion and consensus or, if necessary, by consulting with a third reviewer (YL). In cases where information was missing or unclear, attempts were made to contact the corresponding authors via email. Since no responses were received, all analyses were conducted based on the available published data.

### Risk of bias assessment

Two reviewers (XL and SX) independently assessed the risk of bias in the included studies using the Cochrane Risk of Bias tool (RoB 2.0) for randomized trials, as recommended in the Cochrane Handbook for Systematic Reviews of Interventions (version 6) (41). The following seven domains were evaluated: random sequence generation, allocation concealment, blinding of participants and personnel, blinding of outcome assessment, incomplete outcome data, selective outcome reporting, and other sources of bias. Each domain was rated as having a low, high, or unclear risk of bias. Discrepancies between the reviewers were resolved through discussion and consensus with a third reviewer (YL).

### Statistical analysis

We conducted all meta-analyses following the statistical methods outlined in the Cochrane Handbook for Systematic Reviews of Interventions (version 6). A random-effects model was prespecified as the primary analytical approach for all outcomes to account for anticipated clinical and methodological heterogeneity across studies (e.g., differences in AT protocols, co-interventions, and treatment durations) using Review Manager (RevMan, version 5.3). This approach was applied irrespective of the observed magnitude of statistical heterogeneity. Statistical significance was defined as two-tailed *P* ≤0.05.

Continuous variables were summarized as mean differences (MDs) with 95% confidence intervals (CIs) or standardized mean differences (SMDs) with 95% CIs for outcomes measured using different scales. Categorical variables were reported as risk ratios (RRs) with 95% CIs; odds ratios (ORs) were used as an alternative when the event rates were low.

Statistical heterogeneity was assessed using chi-square test (with *P* < 0.10 indicating statistical significance) and the *I*² statistic. *I*² values were interpreted as follows: 0%–40% represented low heterogeneity, 30%–60% moderate heterogeneity, 50%–90% substantial heterogeneity, and 75%–100% considerable heterogeneity. When *I*² values were close to 0%, fixed-effect and random-effects models are mathematically equivalent and yield nearly identical pooled estimates; therefore, the use of a random-effects model in such cases does not materially influence the direction or magnitude of the results, as discussed in methodological guidance comparing fixed- and random-effects models (42). Consistent results were observed when fixed-effect models were applied as sensitivity analyses (**Supplementary Figure 1**).

To explore potential sources of heterogeneity and further evaluate the effects of AT on PCOS, subgroup analyses (e.g., based on specific AT methods, control interventions, or treatment durations) were performed. Publication bias was assessed using funnel plots for outcomes with at least 10 included studies, with Egger’s or Begg’s tests conducted as appropriate. We also performed sensitivity analyses to examine the robustness of the findings by removing one study at a time and restricting the analysis to studies with a low risk of bias. Forest plots were constructed to visualize the magnitude and direction of treatment effects.

### Assessment of the certainty of the evidence using the GRADE approach

The certainty of the evidence for all outcomes considered critical or important for clinical decision-making was assessed using the Grading of Recommendations, Assessment, Development, and Evaluation (GRADE) approach, as recommended by the GRADE Working Group (43).

Two reviewers (XL and SX) independently assessed the certainty of evidence for each outcome. The certainty was initially considered “high” and subsequently downgraded based on evaluations in five key domains: risk of bias (as assessed by RoB 2.0), inconsistency (heterogeneity), indirectness, imprecision, and publication bias. Any disagreements between the two reviewers were resolved through consensus or, if necessary, by consulting a third reviewer (YL). The overall certainty of the evidence for each outcome was ultimately classified as high, moderate, low, or very low. The results, including the estimated effects and their corresponding certainty ratings, are summarized in the Summary of Findings (SoF) tables.

## Results

### Study selection

The study selection process is illustrated in **Figure 1**. A systematic search identified 504 studies, of which 359 remained after duplicates were removed. During screening of titles and abstracts, 69 studies were deemed potentially relevant, and their full texts were retrieved for further assessment. Subsequently, a total of 47 studies were excluded for the following reasons: non-RCTs (*n* = 6), failure to meet the intervention criteria (*n* = 37), and lack of outcome data (*n* = 4). In total, 18 RCTs met the eligibility criteria and were included in the systematic review and meta-analysis.

### Study characteristics

The primary characteristics of the included studies are summarized in **Tables 1**, **2** provides a summary of auricular acupoint application in the included studies. This systematic review included 18 RCTs (1,231 participants) from mainland China, published between 2011 and 2024. The mean age of participants ranged from 15.89 to 36.89 years, with a mean BMI ranging from 21.37 to 29.52 kg/m² across the studies. Intervention durations ranged from 1 to 6 months.

**Table 1:** Main characteristics of all studies included in the meta-analysis.

Author (year)	Country	PCOS definition criteria	Sample size (I/C)	Age (years, I/C)	BMI (kg/m², I/C)	Intervention	Control	Duration	Outcome indicators	Adverse reaction
Zuo (2011)	China	Rotterdam	20/20	23.80 ± 4.56/24.50 ± 4.37	27.77 ± 2.42/28.00 ± 2.41	AT, 3 times/d + TCMF	TCMF, 2 times/d	3mth	①③④⑤	None
Ling (2015)	China	Rotterdam	36/36	NM/NM	NM/NM	AT, 3 times/d + TCMF	TCMF, 2 times/d	3mth	③④⑤	None
Chen (2021)	China	CMA	37/36	27.78 ± 4.52/28.06 ± 4.27	28.63 ± 3.51/27.92 ± 2.17	AT, 3 times/d + TCMF	TCMF, 2 times/d	3mth	①②③④⑤	None
Wan (2022)	China	CMA	35/36	26.43 ± 4.47/25.75 ± 4.83	22.56 ± 5.27/23.29 ± 2.11	AT, 2 times/d + TCMF	TCMF, 2 times/d	3mth	①④⑤	None
Zhang (2023)	China	Rotterdam	28/28	27.46 ± 4.24/28.49 ± 3.40	27.51 ± 3.94/27.80 ± 3.19	AT, 3 times/d + TCMF	TCMF, 2 times/d	3mth	①②③④⑤⑥⑦⑧⑨⑩	None
Zhu (2023)	China	Rotterdam	25/29	28.70 ± 3.71/28.00 ± 4.16	21.37 ± 1.91/21.61 ± 1.82	AT, 3 times/d + TCMF	TCMF, 2 times/d	6mth	③⑤⑥⑦⑧⑨⑩	None
Gan (2012)	China	Rotterdam	20/20	NM/NM	NM/NM	AT, 3 times/d + MET	MET 500 mg, 3 times/d	3mth	①②	NM
Li (2020)	China	Rotterdam	30/30	29.70 ± 2.60/29.50 ± 2.40	26.30 ± 1.10/26.20 ± 1.00	AT, 3 times/d + MET	MET 500 mg, 3 times/d	3mth	①⑥⑦⑧	NM
Li (2021)	China	CMA	57/57	31.42 ± 3.22/31.48 ± 3.25	NM/NM	AT, 3 times/d + MET	MET 500 mg, 3 times/d	3mth	⑥⑦⑧	NM
Sun (2023)	China	CMA	38/37	30.63 ± 4.22/30.89 ± 3.84	28.27 ± 1.28/27.94 ± 1.28	AT, 4 times/d + MET	MET 500 mg, 3 times/d	3mth	①②③④⑤⑥⑦⑧	None
Zhong (2023)	China	CMA	38/37	15.89 ± 1.72/15.97 ± 1.50	26.95 ± 0.63/27.19 ± 0.55	AT, 4 times/d + MET	MET 500 mg, 3 times/d	3mth	①②③④⑤⑥⑦⑧	None
Zhuang (2023)	China	CMA	39/38	16.03 ± 1.81/15.92 ± 1.67	28.31 ± 1.61/28.24 ± 1.55	AT, 4 times/d + MET	MET 500 mg, 3 times/d	3mth	①②③④⑤	None
Li (2015)	China	Rotterdam	36/36	36.44 ± 3.11/36.89 ± 4.19	23.92 ± 4.19/23.78 ± 5.12	AT, 3-5 times/d + EA	EA, 1 time/W	1mth	③⑦⑧	NM
Liu (2016)	China	Rotterdam	30/28	NM/NM	29.52 ± 3.73/28.31 ± 2.47	AT, 2 times/d + EA	EA, 1 time/2d	3mth	①②	NM
Li (2017)	China	Rotterdam	40/40	27.00 ± 8.00/28.00 ± 7.00	NM/NM	AT, 4 times/d + MA	MA, 1 time/2d	3mth	③④	None
Ma (2017)	China	NM	43/43	35.53 ± 5.18/35.53 ± 5.18	24.53 ± 4.49/24.19 ± 4.57	AT, 3-5 times/d + EA	EA, 1 time/W	1mth	③	NM
Liu (2018)	China	NM	34/34	35.49 ± 4.26/35.44 ± 4.98	24.23 ± 4.66/24.15 ± 4.83	AT, 3-5 times/d + EA	EA, 1 time/W	1mth	③⑧	NM
Zhang (2024)	China	CMA	30/30	28.99 ± 3.58/29.59 ± 3.76	26.06 ± 3.08/26.21 ± 3.25	AT, 1 time/2d + LM	LM	3mth	①⑥⑦⑧⑨⑩	None

TCMF, Traditional Chinese Medicine formula; MET, Metformin; AT, Auricular Therapy; EA, Electric Acupuncture; MA, Manual Acupuncture; LM, Lifestyle Modification; mth, months; w, weeks; d, days; CMA, Chinese Medical Associations; NM, Not Mentioned; ①, BMI; ②, WHR; ③, LH; ④, LH/FSH; ⑤, T; ⑥, FPG; ⑦, FINS; ⑧, HOMA-IR; ⑨, SAS; ⑩, SDS.

**Table 2:** Statistics of auricular acupoint application in the included studies.

Auricular acupoint (corresponding to GB standard number)	Frequency of application	References
CO11 (Uterus)	13/19	Chen S Y 2021, Ling W 2015, Wan X 2022, Zhang W F 2023, Zuo J 2011, Gan L 2012, Li Y C 2020, Li Y C 2021, Li L N 2015, Li Q Q 2017, Liu H J 2016, Liu Y 2018, Ma J J 2017
TG2 (Endocrine)	12/19	Chen S Y 2021, Ling W 2015, Zhang W F 2023, Zhu S Q 2023, Gan L 2012, Li Y C 2020, Li Y C 2021, Li L N 2015, Li Q Q 2017, Liu H J 2016, Liu Y 2018, Ma J J 2017
CO9 (Kidney)	12/19	Chen S Y 2021, Ling W 2015, Wan X 2022, Zhu S Q 2023, Zuo J 2011, Gan L 2012, Li Y C 2020, Li Y C 2021, Sun J G 2023, Zhong L 2023, Zhuang M D 2023, Li L N 2015, Li Q Q 2017, Liu H J 2016, Ma J J 2017
CO4 (Liver)	10/19	Chen S Y 2021, Ling W 2015, Zhang W F 2023, Zhu S Q 2023, Li Y C 2021, Sun J G 2023, Zhong L 2023, Zhuang M D 2023, Li Q Q 2017, Liu H J 2016
CO10 (Spleen)	10/19	Chen S Y 2021, Ling W 2015, Zhang W F 2023, Zuo J 2011, Gan L 2012, Li Y C 2020, Li Y C 2021, Sun J G 2023, Zhong L 2023, Zhuang M D 2023, Li Q Q 2017, Liu H J 2016
AT4 (Subcortex)	8/19	Chen S Y 2021, Ling W 2015, Zhang W F 2023, Zhu S Q 2023, Li Y C 2021, Sun J G 2023, Zhong L 2023, Liu H J 2016
CO12 (Ovary)	8/19	Ling W 2015, Wan X 2022, Zhu S Q 2023, Zuo J 2011, Gan L 2012, Li Y C 2020, Li Y C 2021, Li L N 2015, Li Q Q 2017, Liu H J 2016, Liu Y 2018, Ma J J 2017
CO13 (Stomach)	5/19	Zhang W F 2023, Sun J G 2023, Zhong L 2023, Zhuang M D 2023, Liu H J 2016
TF4 (Shenmen)	4/19	Zhang W F 2023, Zhu S Q 2023, Gan L 2012, Liu H J 2016
TG3 (Hypothalamus)	4/19	Wan X 2022, Li L N 2015, Li Q Q 2017, Liu Y 2018, Ma J J 2017
LO1 (Hunger Point)	4/19	Gan L 2012, Li Y C 2020, Li Y C 2021, Sun J G 2023, Zhong L 2023, Zhuang M D 2023
CO17 (Sanjiao)	4/19	Gan L 2012, Li Y C 2020, Li Y C 2021, Li Q Q 2017
TG1 (Pituitary Gland)	3/19	Wan X 2022, Li L N 2015, Ma J J 2017
CO11 (Internal Genitalia)	3/19	Sun J G 2023, Zhong L 2023, Zhuang M D 2023
HX5 (Edge of Tragus)	2/19	Zhang W F 2023, Zhu S Q 2023
CO7 (Large Intestine)	2/19	Li Y C 2020, Li Y C 2021
AT3 (Brain Point)	1/19	Liu H J 2016
Bilateral Auricular Concha	1/19	Zhang S K 2024

The naming of the auricular acupoints is based on the Chinese National Standard (GB/T 14487-2010). This standard provides clear definitions for the names, locations, and functions of each auricular acupoint. Each acupoint corresponds to a specific code and function, such as CO11 (Uterus) and TG2 (Endocrine). These names are adopted in this study to ensure consistency and scientific accuracy. The functions and codes of the acupoints are aligned with international standards to maintain uniformity and reliability in the research.

The studies investigated various intervention types: AT combined with a TCM formula versus a TCM formula alone (*n* = 6) (44–49), AT plus metformin versus metformin alone (*n* = 6) (38, 50–54), AT plus acupuncture versus acupuncture alone (*n* = 5) (55–59), and AT plus lifestyle modification versus lifestyle modification alone (*n* = 1) (60).

The pre-specified primary outcome was BMI. Secondary outcomes included WHR, LH, LH/FSH ratio, T, FBG, FINS, HOMA-IR, SAS, and SDS. For safety assessments, data on any reported adverse events were systematically extracted and reported.

### Assessment of risk of bias

**Figure 2:**
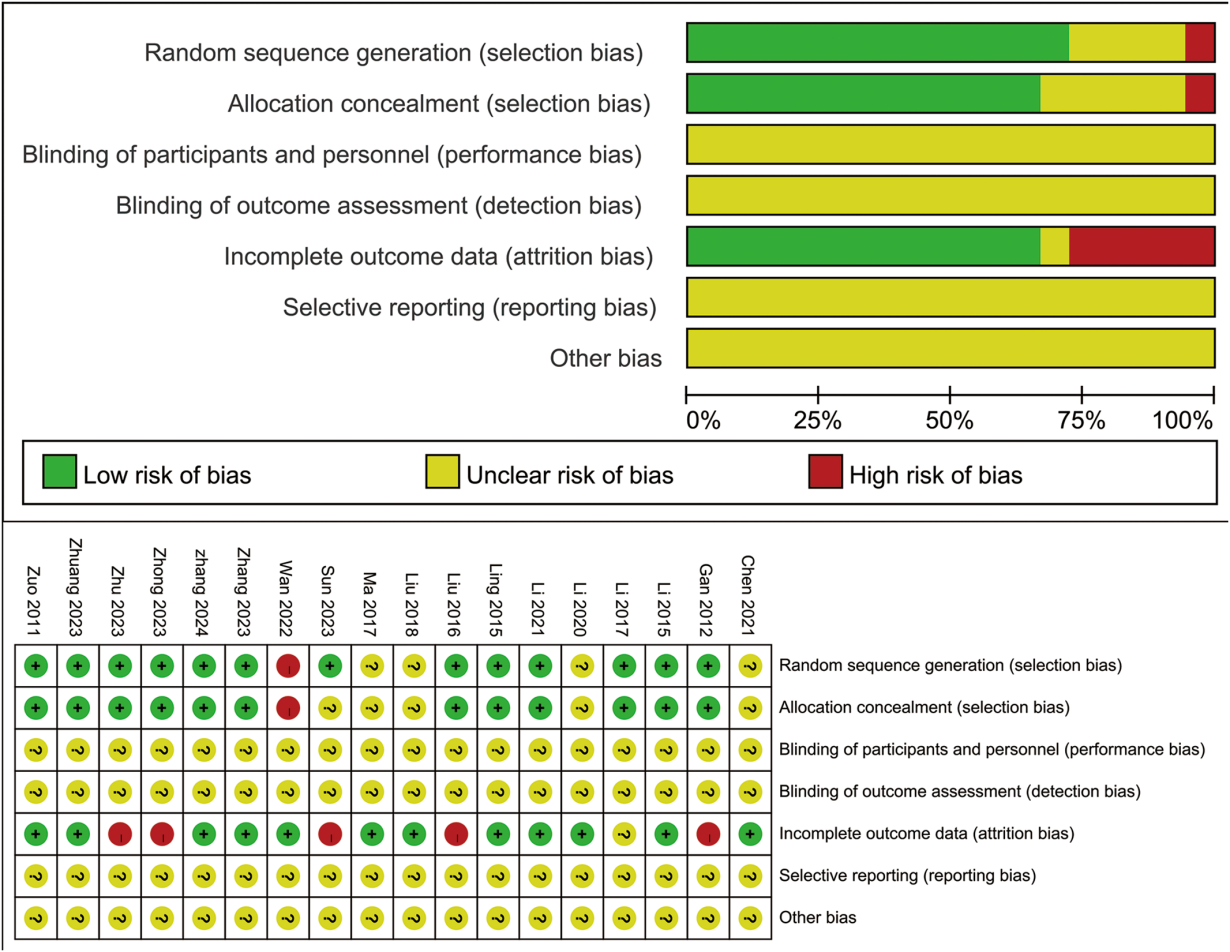
Quality of bias assessment of the included studies.

All 18 included studies were RCTs. Regarding bias arising from the randomization process, 12 studies provided details on sequence generation methods (45, 47–57), while the remaining six did not specify their methods. Allocation concealment was not reported in five studies (38, 44, 52, 58, 59), leading to an unclear risk of bias for this sub-domain. For bias due to missing outcome data, five studies reported participant dropouts without providing reasons for all participants, which resulted in a high-risk rating for this domain (48, 50, 52, 53, 57). Information regarding blinding of participants and personnel (related to bias due to deviations from intended interventions) and blinding of outcome assessors (related to bias in measurement of the outcome) was largely absent across all studies. Similarly, details concerning measurement accuracy for objective outcomes or the transparency of selective outcome reporting were insufficient or not reported. Consequently, the risk of bias due to deviations from intended interventions, measurement bias in the outcome, and bias in the selection of reported result domains was generally judged as “unclear” across most studies due to a lack of information. The risk of bias graph and summary are presented in **Figure 2**.

### Synthesis of results

A total of 18 randomized RCTs, comprising 1,231 women with PCOS, were included in this meta-analysis. The analyses were performed separately for four distinct comparison groups to assess the adjunctive effect of AT. The pooled results for the primary and secondary outcomes are presented below.

#### Effects of AT combined with the TCM formula versus the TCM formula alone on the primary outcome

**Figure 3:**
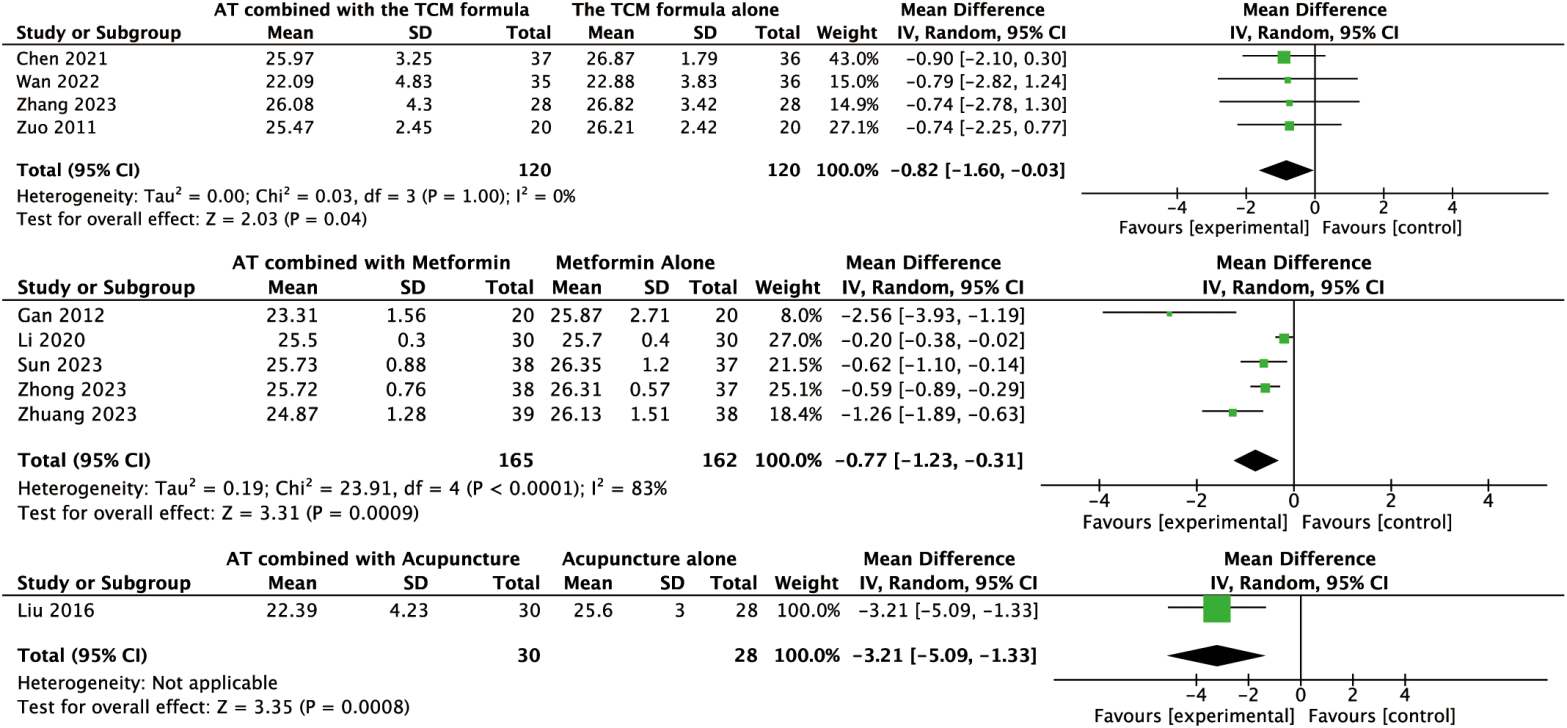
Forest plot displaying the effects of auricular therapy on BMI.

Data for this outcome were available from four trials involving 240 women. A random-effects model was applied to the analysis. AT combined with the TCM formula may improve BMI compared to the TCM formula alone (MD: –0.82, 95% CI: –1.60 to –0.03, *P* = 0.04, I² = 0%, moderate-certainty evidence, **Figure 3**).

#### Effects of AT combined with the TCM formula versus the TCM formula alone on secondary outcomes

**Figure 4:**
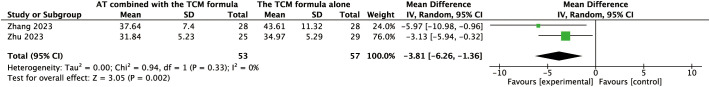
Forest plot displaying the effects of auricular therapy on SAS.

**Figure 5:**
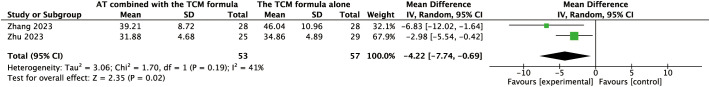
Forest plot displaying the effects of auricular therapy on SDS.

The pooled analysis revealed that AT combined with a TCM formula could reduce SAS (MD: –3.81, 95% CI: –6.26 to –1.36, *P* = 0.002, *I*² = 0%, moderate-certainty evidence, **Figure 4**) and SDS (MD: –4.22, 95% CI: –7.74 to –0.69, *P* = 0.02, *I*² = 41%, moderate-certainty evidence, **Figure 5**) compared to TCM formula alone.

**Figure 6:**
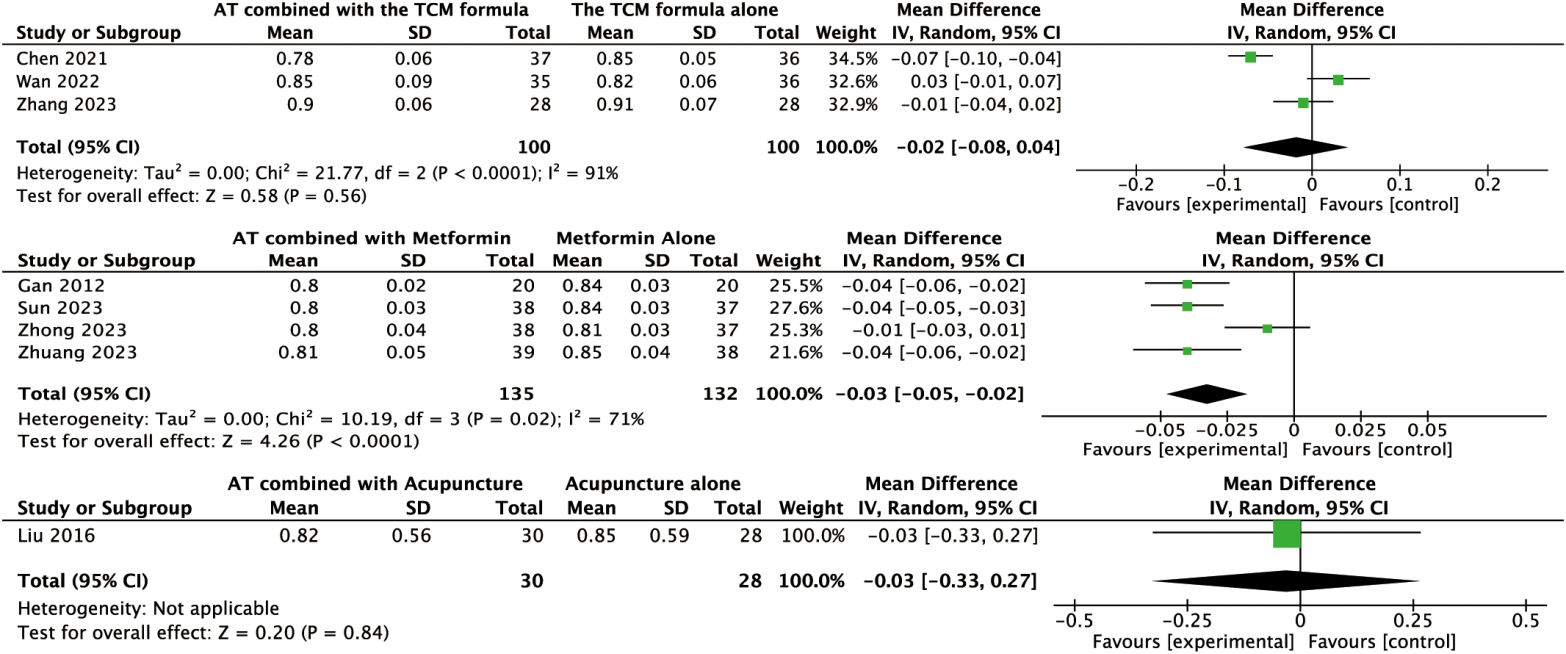
Forest plot displaying the effects of auricular therapy on WHR.

**Figure 7:**
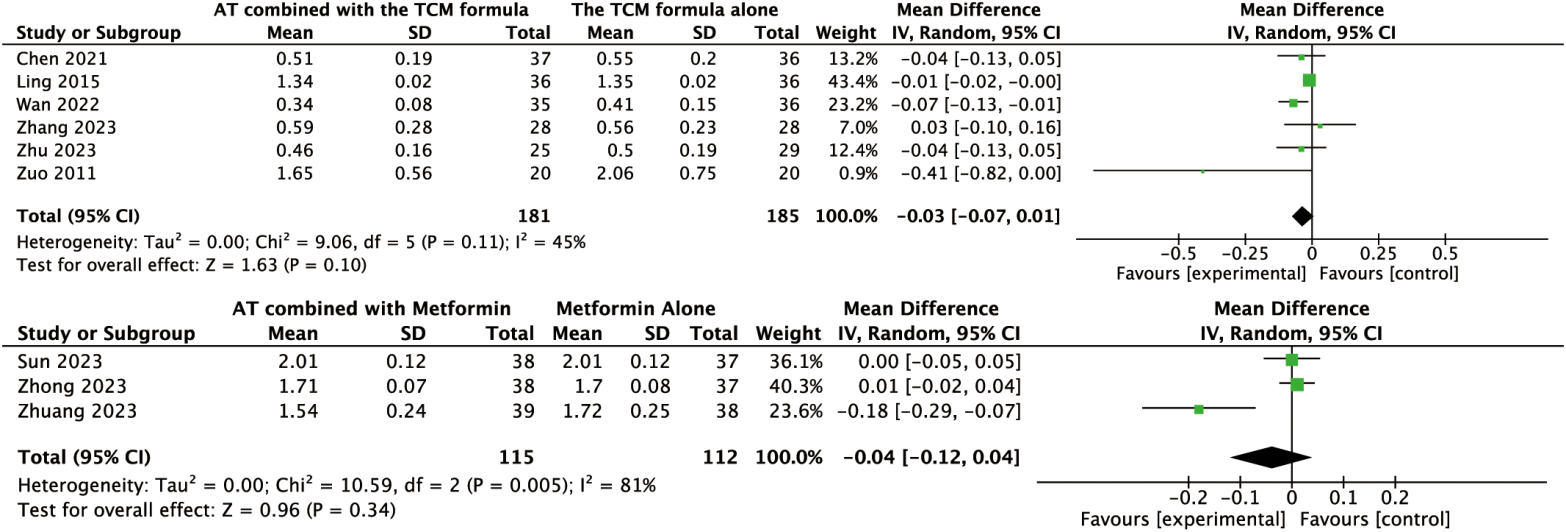
Forest plot displaying the effects of auricular therapy on T.

**Figure 8:**
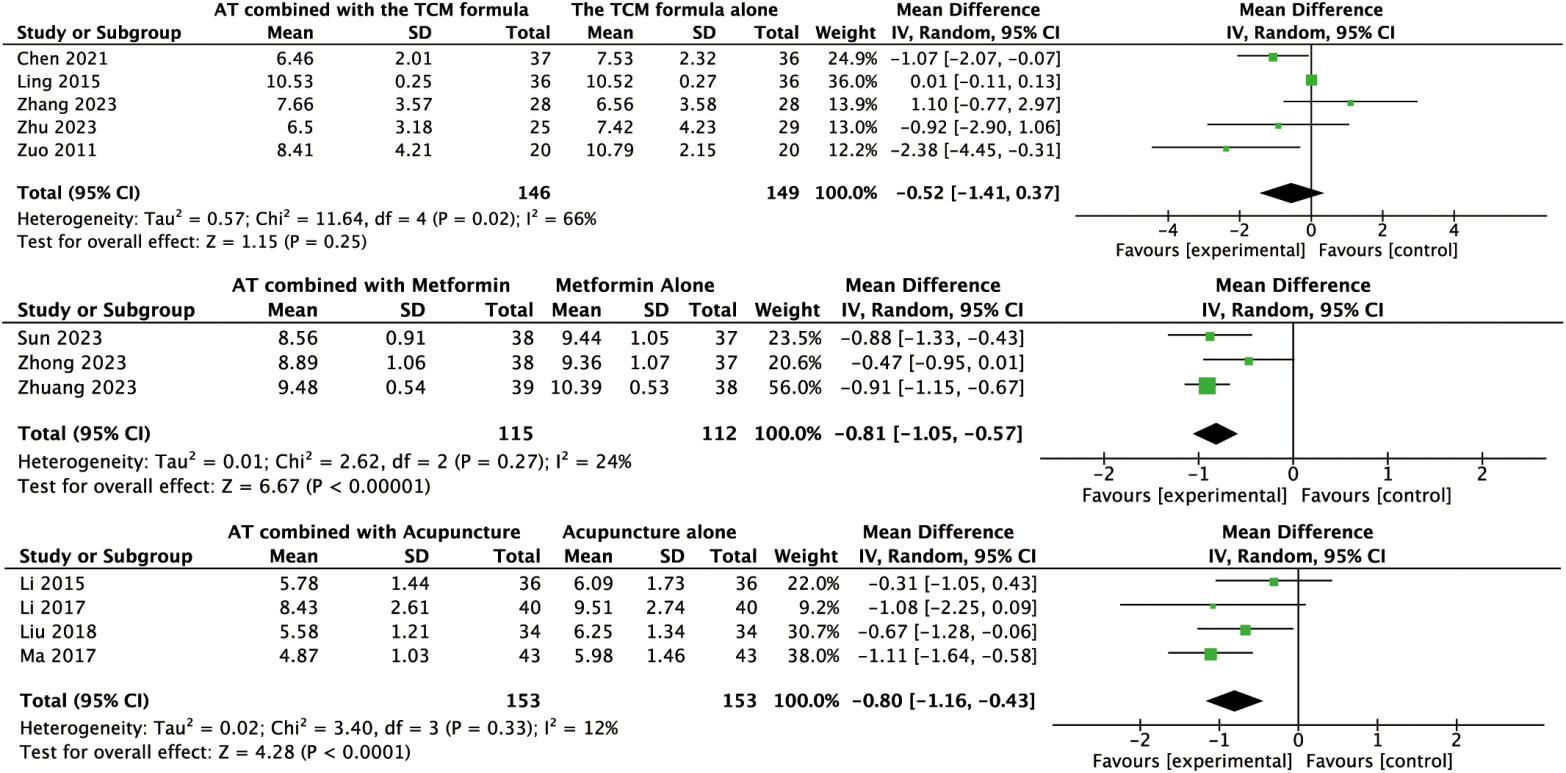
Forest plot displaying the effects of auricular therapy on LH.

**Figure 9:**
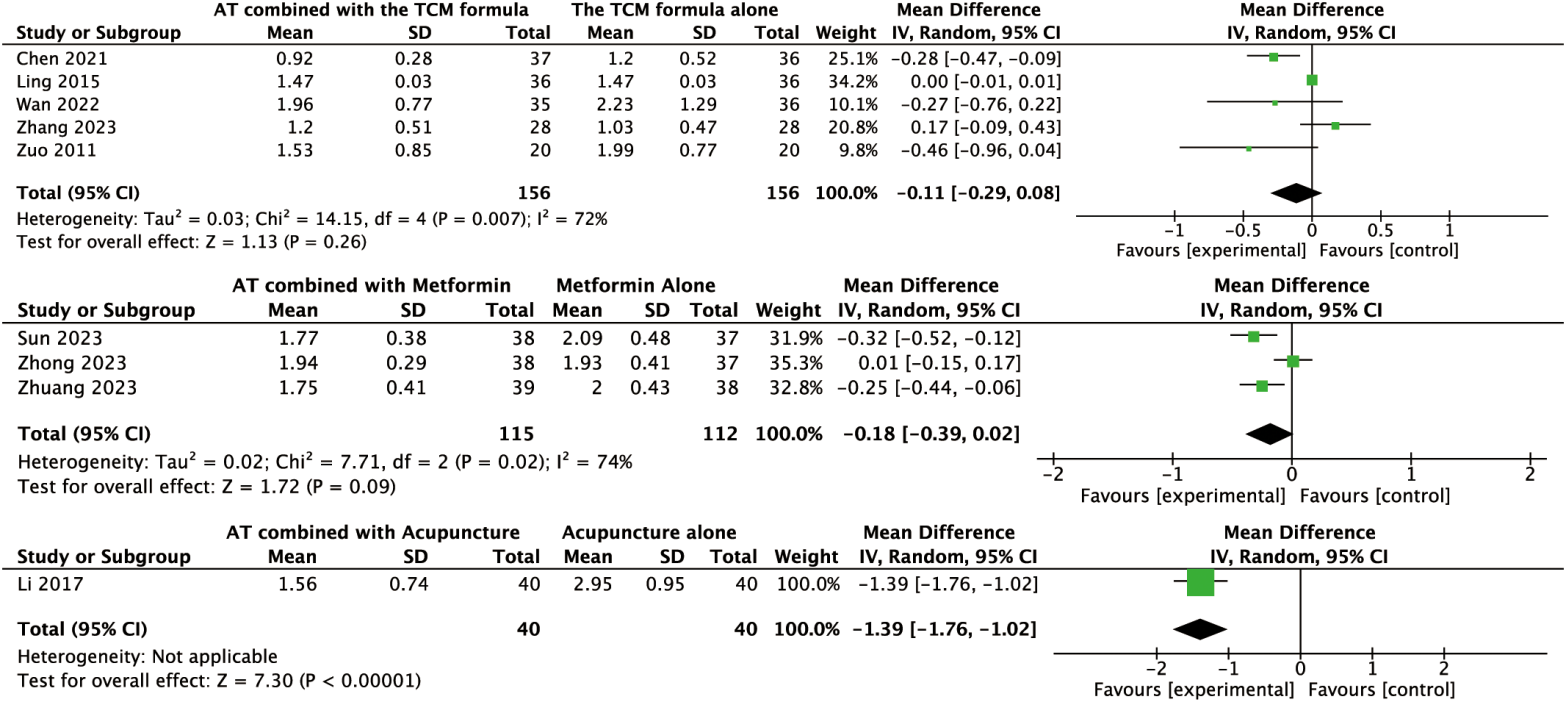
Forest plot displaying the effects of auricular therapy on LH/FSH.

**Figure 10:**
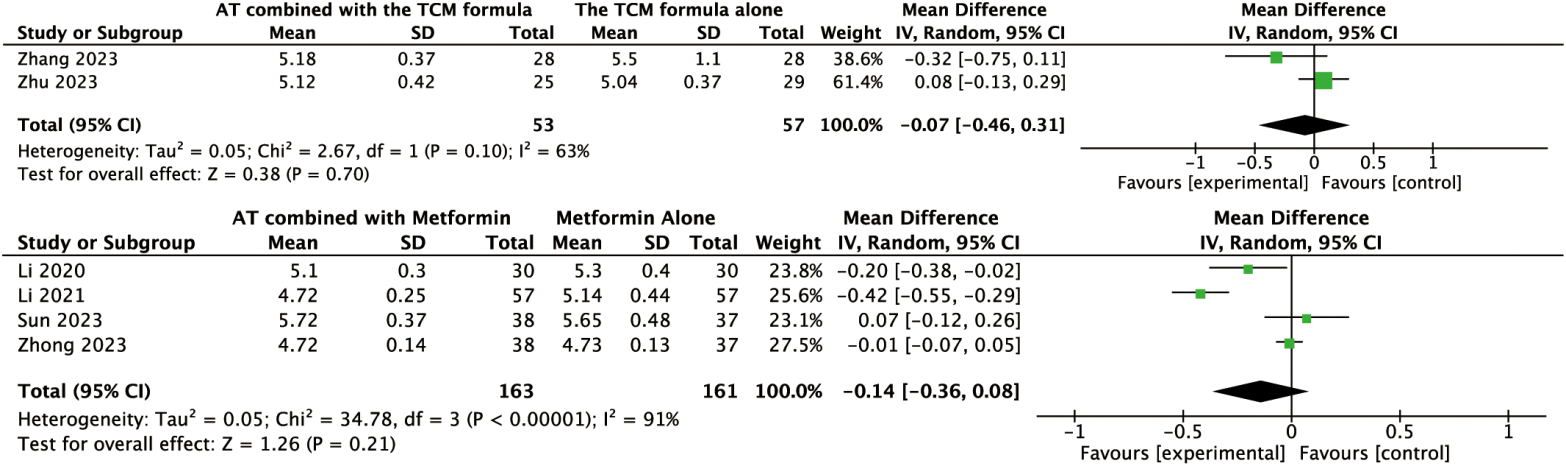
Forest plot displaying the effects of auricular therapy on FBG.

**Figure 11:**
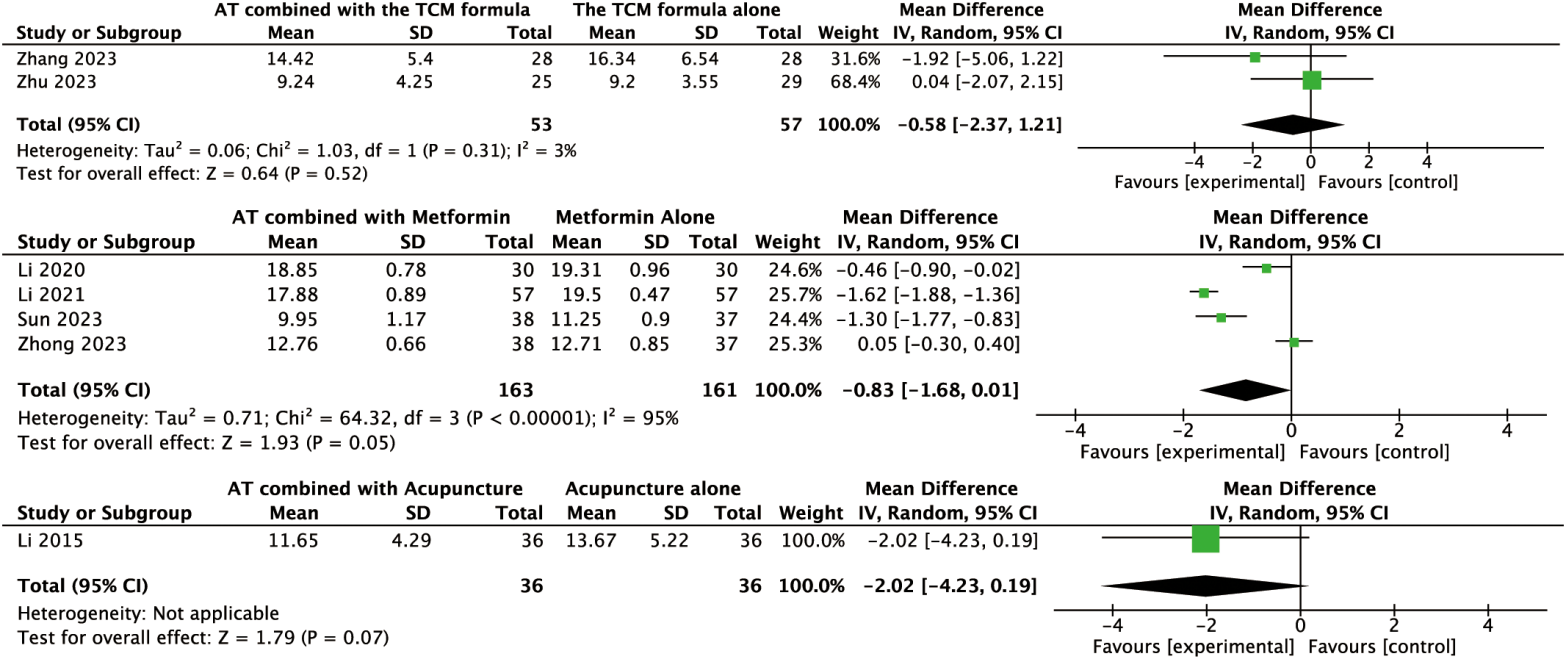
Forest plot displaying the effects of auricular therapy on FINS.

**Figure 12:**
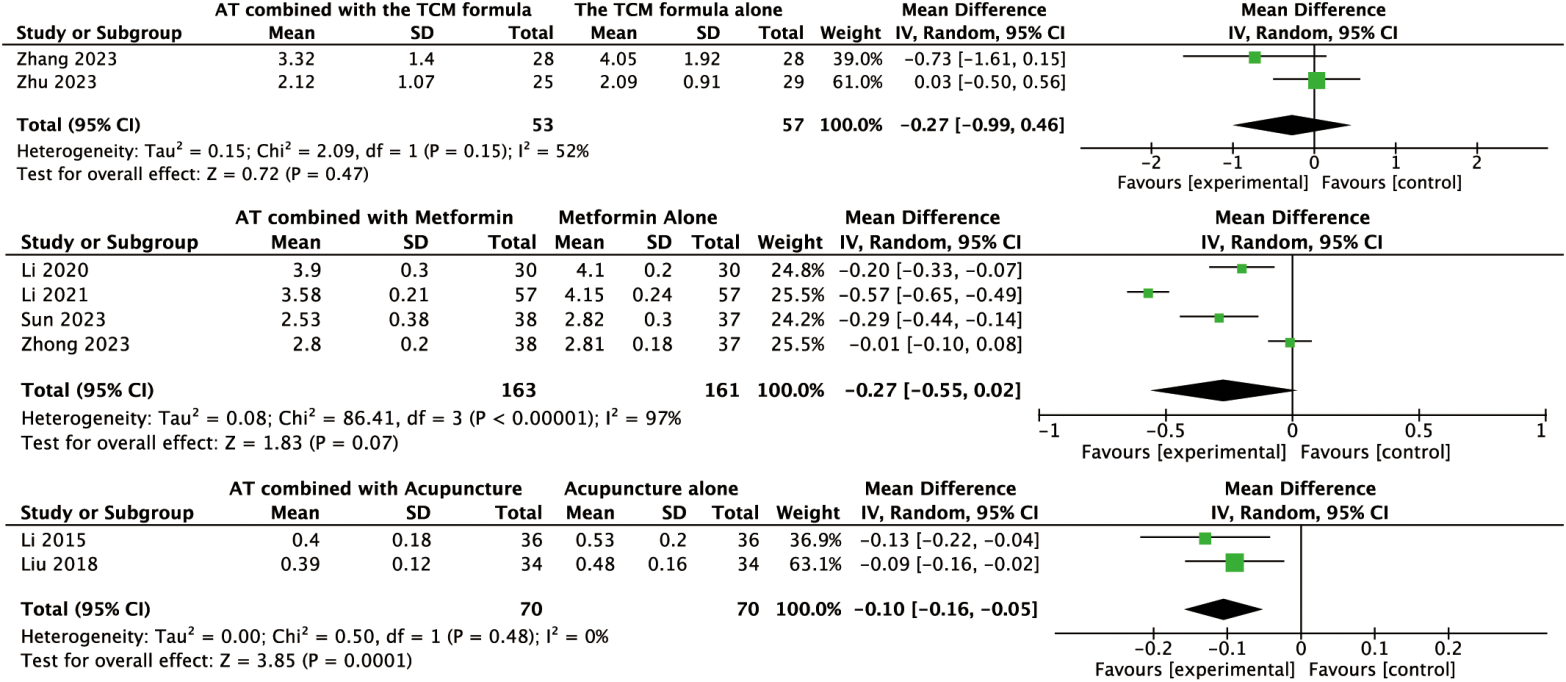
Forest plot displaying the effects of auricular therapy on HOMA-IR.

For other secondary outcomes, the evidence was inconclusive. The analysis showed uncertain effects on WHR (MD: –0.02, 95% CI: –0.08 to 0.04, *P* = 0.56, *I*² = 91%, low-certainty evidence, **Figure 6**) or T levels (MD: –0.03, 95% CI: –0.07 to 0.01, *P* = 0.10, *I*² = 45%, moderate-certainty evidence, **Figure 7**). Similarly, there was no evidence of an effect on LH levels (MD: –0.52, 95% CI: –1.41 to 0.37, *P* = 0.25, *I*² = 66%), the LH/FSH ratio (MD: –0.11, 95% CI: –0.29 to 0.08, *P* = 0.26, *I*² = 72%), FBG (MD: –0.07, 95% CI: –0.46 to 0.31, *P* = 0.70, *I*² = 63%), FINS (MD: –0.58, 95% CI: –2.37 to 1.21, *P* = 0.52, *I*² = 3%), or HOMA-IR (MD: –0.27, 95% CI: –0.99 to 0.46, *P* = 0.47, *I*² = 52%) (**Figures 8**, **9**, **10**–**12**).

#### Effects of AT combined with metformin versus metformin alone on primary outcome

Data for the primary outcome were available from five trials involving 327 women. The pooled results revealed that AT combined with metformin could reduce BMI compared to metformin alone (MD: –0.77, 95% CI: –1.23 to –0.31, *P* < 0.001, *I*² = 83%, low-certainty evidence, **Figure 3**).

#### Effects of AT combined with metformin versus metformin alone on secondary outcomes

Regarding secondary outcomes, the pooled results revealed that combination therapy could reduce WHR (MD: –0.03, 95% CI: –0.05 to –0.02, *P* < 0.001, *I*² = 71%, moderate-certainty evidence, **Figure 6**) and resulted in a reduction of LH levels (MD: –0.81, 95% CI: –1.05 to –0.57, *P* < 0.001, *I*² = 24%, high-certainty evidence, **Figure 8**). However, there was no evidence of an effect on the LH/FSH ratio (MD: –0.18, 95% CI: –0.39 to 0.02, *P* = 0.09, *I*² = 74%, low-certainty evidence, **Figure 9**), T levels (MD: –0.04, 95% CI: –0.12 to 0.04, *P* = 0.34, *I*² = 81%, low-certainty evidence, **Figure 7**), or FBG levels (MD: –0.14, 95% CI: –0.36 to 0.08, *P* = 0.21, *I*² = 91%, low-certainty evidence, **Figure 10**). Furthermore, we found no clear evidence that the combination therapy affected FINS levels (MD: –0.83, 95% CI: –1.68 to 0.01, *P* = 0.05, *I*² = 95%, low-certainty evidence, **Figure 11**) or HOMA-IR (MD: –0.27, 95% CI: –0.55 to 0.02, *P* = 0.07, *I*² = 97%, low-certainty evidence **Figure 12**).

No data were available to determine the effects of AT combined with metformin on SAS or SDS.

#### Effects of AT combined with acupuncture versus acupuncture alone on the primary outcome

Data for the primary outcome were available from a single trial involving 58 women. This study revealed that AT combined with acupuncture could reduce BMI compared to acupuncture alone (MD: –3.21, 95% CI: –5.09 to –1.33, *P* < 0.001, moderate-certainty evidence, **Figure 3**).

#### Effects of AT combined with acupuncture versus acupuncture alone on secondary outcomes

Regarding secondary outcomes, the pooled analysis showed that the combination of AT and acupuncture could reduce LH levels (MD: –0.80, 95% CI: –1.16 to –0.43, *P* < 0.001, *I*² = 12%, high-certainty evidence, **Figure 8**) and HOMA–IR (MD: –0.10, 95% CI: –0.16 to –0.05, *P* < 0.001, *I*² = 0%, high-certainty evidence, **Figure 12**). A single trial consistently suggested that this combination could reduce the LH/FSH ratio (MD: –1.39, 95% CI: –1.76 to –1.02, *P* < 0.001, moderate-certainty evidence, **Figure 9**). However, evidence from a single trial found was uncertain regarding the effect on WHR (MD: –0.03, 95% CI: –0.33 to 0.27, *P* = 0.84, low-certainty evidence, **Figure 6**) and found no evidence of an effect on FINS levels (MD: –2.02, 95% CI: –4.23 to 0.19, *P* = 0.07, low-certainty evidence, **Figure 11**).

Due to a lack of reported data, the effects of AT on T and FBG levels, as well as on SAS/SDS, remain indeterminate.

One study investigated the effectiveness of auricular VN electrical stimulation combined with lifestyle intervention for improving negative emotions in patients with PCOS. In this study, 60 patients with PCOS were randomized into an intervention group (*n* = 30) that received both auricular VN electrical stimulation and lifestyle intervention and a control group (*n* = 30) that received lifestyle intervention alone. Both interventions were administered over a 1-month period. The study reported improvements in the intervention group compared to the control group regarding anxiety (SAS scores) and depression (SDS scores) as well as TCM symptom scores, glucose metabolism indicators (FINS, FBG, and HOMA-IR), and inflammatory markers (IL-1, IL-6, and TNF-α) (all *P* < 0.05).

#### Overall risk of bias

The overall risk of bias across the included studies was judged as moderate to high, primarily due to insufficient reporting of key methodological details. Concerns regarding selection bias were noted. Although all 18 studies were described as randomized, six did not specify the method of sequence generation, and five provided no information on allocation concealment, leaving these studies at an unclear risk. A significant deficiency was the universal lack of information on blinding. No study adequately described the blinding of participants, personnel, or outcome assessors. This resulted in a high or unclear risk of performance bias and detection bias across all trials. Attrition bias was a concern in five studies that reported dropouts without providing an adequate explanation for all missing participants, leading to a high-risk judgment in this domain. Finally, insufficient detail regarding selective outcome reporting was a common issue, making it challenging to assess the risk of bias in reporting. Due to this widespread lack of information, many domains were frequently rated as having an “unclear risk of bias”. A detailed summary of the risk of bias assessment for each study is presented in **Figure 2**.

#### Reporting biases

The risk of reporting bias was high or unclear across all studies, given that no trial provided evidence of a pre-registered study protocol. The absence of accessible protocols made it challenging to verify whether the reported outcomes were consistent with a predefined analysis plan, raising concerns about potential selective reporting and outcome switching.

The potential for publication bias was also assessed. Due to the small number of studies (<10) in most meta-analyses, the use of funnel plots to detect asymmetry was not feasible for most outcomes. For the few outcomes where analysis was possible, visual inspection of the funnel plots suggested some asymmetry, which may indicate publication bias. However, these findings should be interpreted with caution, as the statistical power to detect true bias was limited.

These identified risks of bias were considered in the GRADE assessment and contributed to the downgrading of the certainty of evidence for several outcomes.

#### Certainty of the evidence

The certainty of the evidence for all outcomes was evaluated using the GRADE approach. The overall certainty ranged from high to low across the comparisons, with downgrading primarily due to serious risk of bias, inconsistency across studies, and potential publication bias.

A comprehensive summary of these GRADE assessments, detailing the reasons for each rating, is presented in the Summary of Findings table (**Supplementary Table 1**).

#### Robustness of findings

Sensitivity analyses were performed to assess the robustness of our findings. Despite the methodological limitations identified in the included studies, excluding studies with a high risk of bias did not substantially alter the overall direction or magnitude of the pooled estimates. This finding suggests a certain level of consistency and reliability in the therapeutic effect despite the overall low-to-moderate certainty of the evidence.

## Discussion

Emerging evidence highlights PCOS as a neuroendocrine condition (61, 62), with its pathogenesis closely linked to dysregulation of the hypothalamic–pituitary–ovarian (HPO) axis, hypothalamic–pituitary–adrenal (HPA) axis, and, increasingly, autonomic nervous system (ANS) dysfunction (63). This growing recognition of the ANS’ role underscores the complexity of PCOS etiology and suggests that interventions targeting the nervous system may hold therapeutic potential (64–67).

To the best of our knowledge, this systematic review and meta-analysis is the first to systematically evaluate the efficacy and safety of AT for managing PCOS. Given the limited evidence available for AT as a standalone treatment, our analysis focused on AT as an adjunctive therapy combined with other interventions.

Our findings demonstrated that AT, when used in combination with conventional or complementary treatments, showed beneficial effects on anthropometric, hormonal, metabolic, and psychological outcomes. BMI showed improvement across all three intervention groups, while WHR was reduced when AT was combined with metformin. The LH levels were reduced when AT was combined with metformin and acupuncture, and the LH/FSH ratio decreased in the AT combined with acupuncture group. Besides that, HOMA-IR was reduced in the AT combined with acupuncture group. Psychological outcomes, as assessed by SAS and SDS, also showed significant improvement when AT was combined with TCM formulas.

Despite these promising findings, the results of this study should be interpreted with caution. Our analysis revealed several methodological limitations across the included RCTs, which may compromise the reliability and validity of the findings. Specifically, most studies were characterized by small sample sizes, inadequate reporting of key methodological details (such as allocation concealment and blinding), and a high risk of selective reporting bias. These issues were the primary reasons for the downgrading of evidence certainty as assessed by the GRADE approach. Across different outcomes, the certainty of evidence ranged from low to high, reflecting serious concerns regarding risk of bias, inconsistency, and potential publication bias in the primary studies. These factors reduce confidence in the estimated effects of AT for PCOS.

In addition, considerable heterogeneity was observed across the included studies. A random-effects model was employed to minimize its impact, and subgroup and sensitivity analyses were conducted. The subgroup analyses were performed based on different intervention methods, and RCTs with small sample sizes or a high risk of bias were temporarily excluded from the primary analysis to assess the robustness of the findings. The sensitivity analyses indicated that excluding these studies did not substantially alter the overall results; accordingly, they were retained in the final meta-analysis.

Nevertheless, heterogeneity persisted despite these efforts and may be attributed to several factors. Initially, some trials lacked sufficient information on participant characteristics, such as BMI and age, thereby limiting accurate subgroup stratification. Second, repeated subgroup analyses may increase the likelihood of type I errors, potentially leading to false-positive findings. More importantly, substantial heterogeneity was observed in certain outcome comparisons, particularly when AT was combined with different co-interventions—for example, the heterogeneity for BMI outcomes was minimal when AT was combined with the TCM formula (*I*² = 0%), whereas marked heterogeneity emerged when AT was combined with metformin (*I*² = 83%). This discrepancy suggests that the observed heterogeneity is likely driven by variations in the co-interventions combined with AT and thus contributes substantially to between-study variability. In addition, the included studies varied considerably in terms of baseline disease severity, intervention duration, concurrent medication use, and specific AT protocols (e.g., auricular acupuncture versus auricular acupressure, stimulation frequency, and point selection). Such heterogeneity in clinical characteristics and trial design may be further amplified in the context of adjunctive therapy. These methodological inconsistencies likely contributed to the observed variability and should be carefully considered when interpreting the findings of this meta-analysis. Moreover, this review included only studies published in Chinese, which may have introduced language and publication bias, thereby limiting the generalizability and strength of the conclusions.

An increasing body of evidence suggests that dysfunction of the ANS, particularly increased sympathetic activity alongside reduced parasympathetic activity, may contribute to PCOS development and progression (64–67). Under normal physiological conditions, the sympathetic and parasympathetic systems operate in a dynamic balance to maintain homeostasis. Disruption of this balance has been associated with various disorders involving autonomic dysregulation, including neurological, metabolic, inflammatory, cardiovascular, and psychiatric conditions (68). Over the years, several studies have indicated that women with PCOS exhibit increased sympathetic activity, and a complex bidirectional relationship may exist between sympathetic overactivity and endocrine or metabolic disturbances (64, 69–71). These observations have led to the hypothesis that therapeutic strategies aimed at reducing sympathetic activity or enhancing parasympathetic function, thereby restoring sympathovagal balance, may be beneficial in managing PCOS-related symptoms (18).

**Figure 13:**
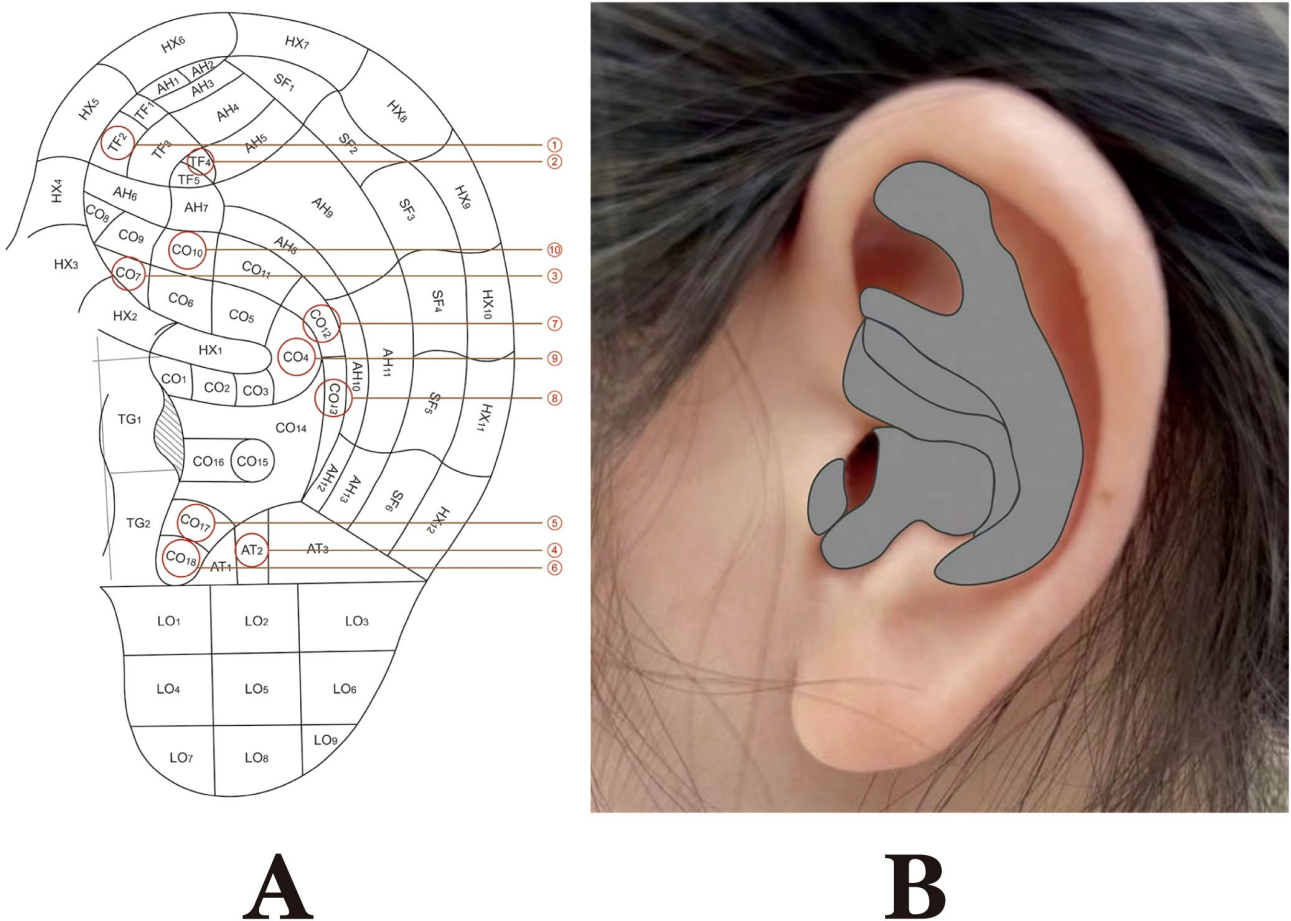
Auricular acupoints and the distribution area of the auricular vagus nerve. **(A)** Numbers 1 through 10 correspond to the locations of auricular acupuncture points used in the articles, as depicted on the national standard auricular map. Specifically, the auricular points for numbers 1, 2, 3, 4, 5, 6, 7, 8, 9, and 10 are as follows: CO11 (internal genitalia), TF4 (shenmen), CO7 (large intestine), AT4 (subcortex), CO17 (sanjiao), TG2 (endocrine), CO12 (liver), CO13 (spleen), CO4 (stomach), and CO10 (kidney). **(B)** The gray-shaded area represents the region innervated by the ABVN.

AT integrates the theoretical foundations of TCM with modern neuroanatomical understanding, with its core mechanism believed to involve the stimulation of the auricular branch of the VN (ABVN). The auricle is the peripheral body region innervated by the ABVN (72, 73), as illustrated in **Figure 13**. It is now understood that the ABVN projects to key nuclei in the brainstem, including the nucleus tractus solitarius (NTS) and the dorsal motor nucleus (DMN) of the vagus, forming the neuroanatomical basis of the auricle–vagal reflex (74). This anatomical arrangement provides a plausible rationale for modulating physiological functions through the targeted stimulation of specific auricular acupoints.

In the context of PCOS, the commonly used auricular points, such as TG2 (endocrine), TF4 (shenmen), CO11 (internal genitalia), and CO10 (kidney), are located within the ABVN-innervated regions of the auricle (75). Details on the selection of auricular acupoints and their frequency patterns when combined with other interventions are provided in **Table 2**.

The anatomical distribution of the auricular acupoints is depicted in **Figure 13**. It has been proposed that ta-VNS targeting these regions may activate central vagal pathways, potentially contributing to autonomic regulation and systemic homeostasis (76). Findings from the present meta-analysis suggest that AT, particularly ta-VNS, may play a role in alleviating autonomic dysfunction in PCOS by modulating the ABVN–NTS/DMN axis. While the exact mechanisms remain fully elucidated, preliminary evidence supports the involvement of multiple integrative pathways, warranting further investigation.

**Figure 14:**
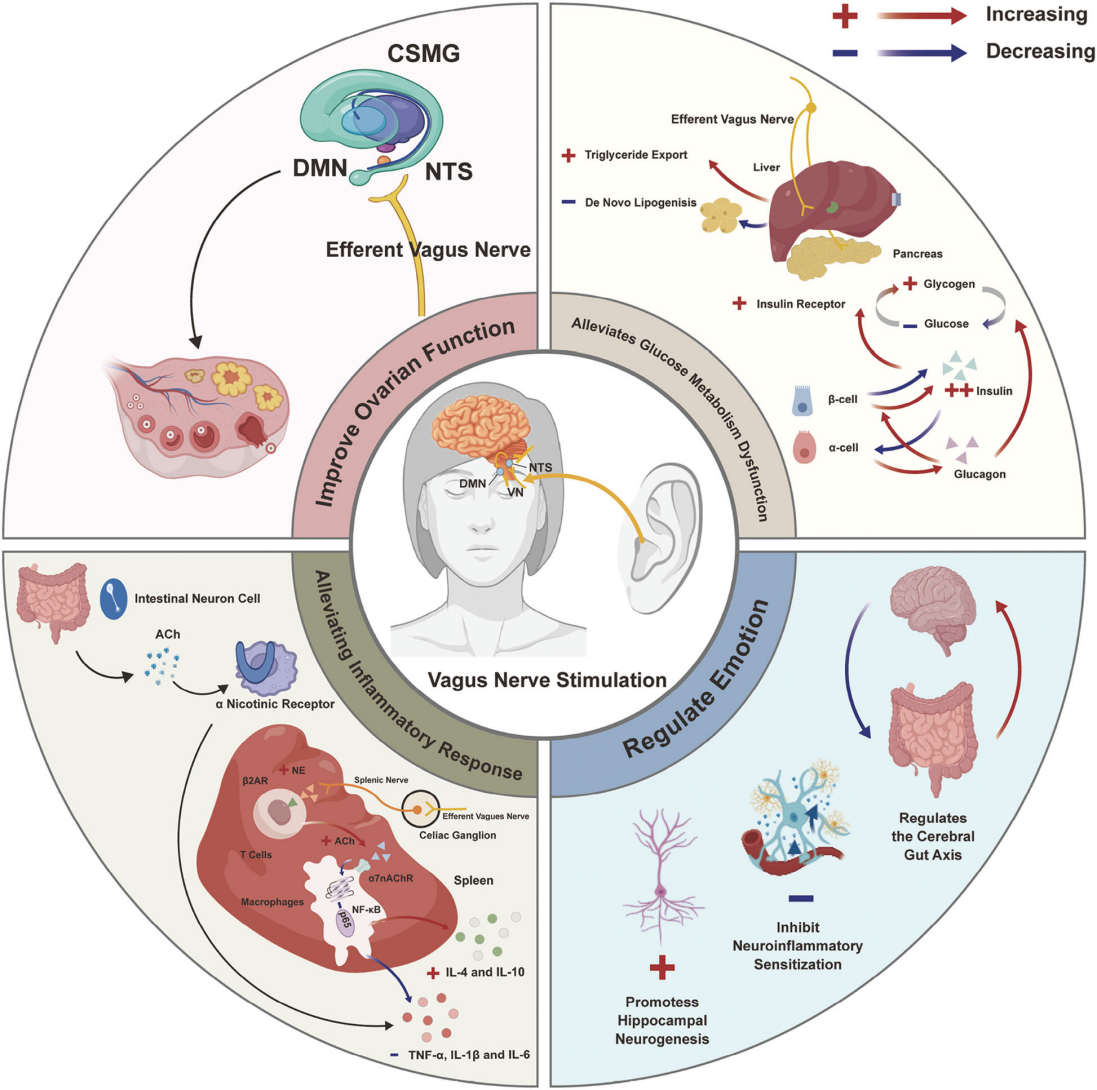
Potential mechanisms of auricular therapy in treating PCOS via vagal nerve stimulation.

From a mechanistic perspective, the therapeutic effects of AT may be mediated through multiple integrative pathways. The underlying schematic diagram is potentially shown in **Figure 14**.

First, AT may modulate the central regulation of ovarian function, thereby improving sex hormone imbalance. Gerendai et al. proposed that a neural circuit may exist between the ovaries and the central nervous system (CNS), whereby the ovaries receive afferent input from the CNS through the superior ovarian nerve (SON), ovarian plexus nerve, and VN and transmit signals back to the CNS through the SON and the celiac–superior mesenteric ganglia, while also responding to afferent vagal input (77, 78). Growing evidence suggests that afferent fibers of the VN may modulate the secretion of gonadotropins by acting on the hypothalamus, thereby modulating hypothalamic control over ovarian function (79, 80). These findings suggest that VNS plays a significant role in regulating ovarian physiology. Although the precise mechanisms by which AT affects ovarian function remain unclear, future studies should explore whether AT modulates the pulsatile secretion of gonadotropin-releasing hormone (GnRH) in the hypothalamus, thereby improving the function of the HPO axis and alleviating the dysregulated LH secretion commonly observed in PCOS.

Second, a key pathophysiological mechanism of PCOS is IR. The VN is widely known to play a vital role in maintaining glucose homeostasis, a process central to IR development. Afferent signals from the ABVN transmit nutritional information to the medulla’s dorsal vagal complex, activating vagal efferent pathways that regulate hepatic gluconeogenesis (81). Furthermore, AT has been shown to improve IR by promoting early-phase insulin secretion and enhancing hepatic insulin sensitivity (82). Therefore, by targeting the vagal pathway, AT may offer a novel approach to directly address IR, a core endocrine abnormality in PCOS.

Third, AT appears to exert anti-inflammatory effects, primarily through the cholinergic anti-inflammatory pathway (CAP) and the splenic sympathetic anti-inflammatory pathway (SSAP) (83–85). There is a growing consensus suggesting that PCOS is characterized by chronic low-grade inflammation, with elevated levels of pro-inflammatory cytokines such as interleukin-6 (IL-6), tumor necrosis factor-α (TNF-α), and C-reactive protein contributing to IR and metabolic dysfunction (86–88). In the CAP, vagal signaling stimulates enteric neurons to release acetylcholine (ACh), which binds to α7 nicotinic ACh receptors (α7nAChR) on macrophages, thereby downregulating the expression of these PCOS-associated pro-inflammatory cytokines. In the SSAP, vagal modulation of visceral sympathetic nerves promotes ACh secretion from T cells, which similarly acts on α7nAChR to inhibit nuclear factor kappa B signaling, a key pathway in PCOS-related inflammation. By targeting these inflammatory pathways, AT may help break the cycle of inflammation-induced IR and hormonal dysregulation that perpetuates PCOS symptoms.

Fourth, AT may contribute to metabolic regulation and weight management, addressing key pathophysiological features of PCOS. Obesity affects 50%–80% of PCOS patients and exacerbates IR, hyperandrogenism, and reproductive dysfunction. Evidence suggests that AT can reduce postprandial ghrelin levels and suppress appetite, increase basal metabolic rate, and promote visceral fat consumption, effects that target the metabolic dysfunction characteristic of PCOS (89–94).

Fifth, emerging evidence suggests that AT may potentially help alleviate psychological symptoms commonly observed in patients with PCOS, including depression and anxiety, though the underlying mechanisms and clinical efficacy require further investigation. Proposed mechanisms may involve modulation of neural activity and connectivity in depression-related brain regions (95), inhibition of neuroinflammatory sensitization (95), promotion of hippocampal neurogenesis (96), and regulation of the gut–microbiota–brain axis (97).

In summary, AT demonstrates broad therapeutic potential in managing PCOS through its multi-target and multi-mechanism regulatory effects. Its neurophysiological basis provides a preliminary foundation for further clinical application. Although preliminary evidence supports the effectiveness of AT in treating PCOS, the underlying neurobiological mechanisms remain to be fully elucidated. Future studies should systematically investigate the multi-level neural regulatory pathways involved, particularly by utilizing advanced neuroimaging techniques such as functional magnetic resonance imaging and diffusion tensor imaging, to explore how ta-VNS modulates brain networks, neural circuits, and neurotransmitter metabolism.

Future research directions should prioritize two critical domains. First, mechanistic investigations must elucidate whether ta-VNS modulates hypothalamic GnRH pulsatility to restore HPO axis function and normalize LH secretion patterns in PCOS. This hypothesis warrants an investigation of central neuroendocrine pathways and their downstream effects on ovarian function, potentially establishing AT as a precision neuromodulation therapy targeting the core pathophysiology of PCOS.

Second, the standardization and optimization of AT protocols demand immediate attention. Future studies must establish evidence-based parameters, including optimal acupoint selection (e.g., TF4 (shenmen), TG2 (endocrine)), stimulation intensity (typically 2–4 mA for electroacupuncture), frequency specifications (10–100 Hz), treatment duration (20–30 min per session), intervention cycles (8–12 weeks), and maintenance protocols. Besides that, determining patient-specific factors such as BMI-adjusted dosing, phenotype-specific acupoint combinations, and biomarker-guided treatment timing will advance AT toward precision medicine applications.

Indeed large-scale, multicenter RCTs employing these standardized protocols are essential to establish clinical efficacy and safety profiles. These trials should incorporate rigorous dose–response analyses, long-term follow-up assessments, and investigation of AT synergies with conventional PCOS therapies. Such a comprehensive research framework will facilitate AT’s evolution from empirical practice to evidence-based, mechanism-driven therapeutic strategy, ultimately expanding treatment options for diverse PCOS phenotypes.

## Conclusions

This systematic review and meta-analysis demonstrated that AT could serve as a beneficial adjunctive treatment for PCOS management. Our findings indicated that AT reduced BMI and improved LH, LH/FSH ratio, HOMA-IR, and anxiety and depression scores. No serious adverse events were reported; however, this finding requires cautious interpretation due to potential underreporting and limited follow-up periods in primary studies.

Several methodological limitations constrain these conclusions, including small sample sizes, heterogeneity in intervention protocols, and limited study duration. These limitations necessitate larger, well-designed RCTs with standardized protocols to establish AT’s long-term efficacy and safety profile in PCOS management.

Future research priorities should include mechanistic investigations of AT’s neuromodulatory pathways, particularly whether transcutaneous VN stimulation modulates hypothalamic GnRH plasticity and the function of the HPO axis. Moreover, standardization of treatment parameters, including optimal acupoint selection, stimulation intensity, frequency, and treatment duration, is essential for clinical translation.

## Data Availability

The original contributions presented in the study are included in the article/**Supplementary Material**. Further inquiries can be directed to the corresponding author.

## Supplementary material

Supplementary Material 1 PRISMA checklist.

Supplementary Material 2 Full search strategy.

Supplementary Table 1 S3 Summary of Findings.

## References

[1] Iervolino M Lepore E Forte G Laganà AS Buzzaccarini G Unfer V . Natural molecules in the management of polycystic ovary syndrome (PCOS): an analytical review. Nutrients. (2021) 13. doi: 10.3390/nu13051677, PMID: 34063339 PMC8156462

[2] Mimouni NEH Paiva I Barbotin AL Timzoura FE Plassard D Le Gras S . Polycystic ovary syndrome is transmitted via a transgenerational epigenetic process. Cell Metab. (2021) 33:513–30.e8. doi: 10.1016/j.cmet.2021.01.004, PMID: 33539777 PMC7928942

[3] Yang R Li Q Zhou Z Qian W Zhang J Wu Z . Changes in the prevalence of polycystic ovary syndrome in China over the past decade. Lancet Reg Health West Pac. (2022) 25:100494. doi: 10.1016/j.lanwpc.2022.100494, PMID: 35669932 PMC9162959

[4] Joham AE Boyle JA Zoungas S Teede HJ . Hypertension in reproductive-aged women with polycystic ovary syndrome and association with obesity. Am J Hypertens. (2015) 28:847–51. doi: 10.1093/ajh/hpu251, PMID: 25542625

[5] Joham AE Norman RJ Stener-Victorin E Legro RS Franks S Moran LJ . Polycystic ovary syndrome. Lancet Diabetes Endocrinol. (2022) 10:668–80. doi: 10.1016/s2213-8587(22)00163-2, PMID: 35934017

[6] Teede HJ Misso ML Costello MF Dokras A Laven J Moran L . Recommendations from the international evidence-based guideline for the assessment and management of polycystic ovary syndrome. Hum Reprod. (2018) 33:1602–18. doi: 10.1093/humrep/dey256, PMID: 30052961 PMC6112576

[7] Choudhari R Tayade S Tiwari A Satone P . Diagnosis, management, and associated comorbidities of polycystic ovary syndrome: A narrative review. Cureus. (2024) 16:e58733. doi: 10.7759/cureus.58733, PMID: 38779261 PMC11110474

[8] Cowan S Lim S Alycia C Pirotta S Thomson R Gibson-Helm M . Lifestyle management in polycystic ovary syndrome - beyond diet and physical activity. BMC Endocr Disord. (2023) 23:14. doi: 10.1186/s12902-022-01208-y, PMID: 36647089 PMC9841505

[9] Meier RK . Polycystic ovary syndrome. Nurs Clin North Am. (2018) 53:407–20. doi: 10.1016/j.cnur.2018.04.008, PMID: 30100006

[10] Prelević GM Würzburger MI Balint-Perić L Puzigaća Z . Effects of a low-dose estrogen-antiandrogen combination (Diane-35) on clinical signs of androgenization, hormone profile and ovarian size in patients with polycystic ovary syndrome. Gynecol Endocrinol. (1989) 3:269–80. doi: 10.3109/09513598909152466, PMID: 2516704

[11] Flory J Lipska K . Metformin in 2019. JAMA. (2019) 321:1926–7. doi: 10.1001/jama.2019.3805, PMID: 31009043 PMC7552083

[12] Malik S Saeed S Saleem A Khan MI Khan A Akhtar MF . Alternative treatment of polycystic ovary syndrome: pre-clinical and clinical basis for using plant-based drugs. Front Endocrinol (Laus). (2023) 14:1294406. doi: 10.3389/fendo.2023.1294406, PMID: 38725974 PMC11081130

[13] Verma A Upadhyay V Saxena V . Effect of yoga therapy on health outcomes in women with polycystic ovary syndrome: A systematic review and meta-analysis. Am J Lifesty Med. (2023) 17:73–92. doi: 10.1177/15598276211029221, PMID: 36636398 PMC9830238

[14] Zhao K Nie L Ye X Hu X . Effects of mind-body interventions on polycystic ovary syndrome: a comprehensive meta-analysis. J Ovarian Res. (2024) 17:154. doi: 10.1186/s13048-024-01477-2, PMID: 39054488 PMC11271059

[15] Ye Y Zhou CC Hu HQ Fukuzawa I Zhang HL . Underlying mechanisms of acupuncture therapy on polycystic ovary syndrome: Evidences from animal and clinical studies. Front Endocrinol (Laus). (2022) 13:1035929. doi: 10.3389/fendo.2022.1035929, PMID: 36353235 PMC9637827

[16] Li Y Zheng X Wang Y Li Y . Auricular therapy for polycystic ovary syndrome: A protocol for a systematic review and meta-analysis. Med (Balt). (2020) 99:e23396. doi: 10.1097/md.0000000000023396, PMID: 33285726 PMC7717724

[17] Teede HJ Tay CT Laven JJE Dokras A Moran LJ Piltonen TT . Recommendations from the 2023 international evidence-based guideline for the assessment and management of polycystic ovary syndrome. J Clin Endocrinol Metab. (2023) 108:2447–69. doi: 10.1210/clinem/dgad463, PMID: 37580314 PMC10505534

[18] Zhang S He H Wang Y Wang X Liu X . Transcutaneous auricular vagus nerve stimulation as a potential novel treatment for polycystic ovary syndrome. Sci Rep. (2023) 13:7721. doi: 10.1038/s41598-023-34746-z, PMID: 37173458 PMC10182028

[19] Nogier R . History of auriculotherapy: additional information and new developments. Med Acupunct. (2021) 33:410–9. doi: 10.1089/acu.2021.0075, PMID: 34976274 PMC8716479

[20] Wirz-Ridolfi A . The history of ear acupuncture and ear cartography: why precise mapping of auricular points is important. Med Acupunct. (2019) 31:145–56. doi: 10.1089/acu.2019.1349, PMID: 31297168 PMC6604909

[21] Yu Y Chen T Zheng Z Jia F Liao Y Ren Y . The role of the autonomic nervous system in polycystic ovary syndrome. Front Endocrinol (Laus). (2023) 14:1295061. doi: 10.3389/fendo.2023.1295061, PMID: 38313837 PMC10834786

[22] Lin YW Hsieh CL . Auricular electroacupuncture reduced inflammation-related epilepsy accompanied by altered TRPA1, pPKCα, pPKCϵ, and pERk1/2 signaling pathways in kainic acid-treated rats. Mediators Inflammation. (2014) 2014:493480. doi: 10.1155/2014/493480, PMID: 25147437 PMC4131505

[23] Zhao YX He W Jing XH Liu JL Rong PJ Ben H . Transcutaneous auricular vagus nerve stimulation protects endotoxemic rat from lipopolysaccharide-induced inflammation. Evid Based Complement Alternat Med. (2012) 2012:627023. doi: 10.1155/2012/627023, PMID: 23346208 PMC3544369

[24] Romoli M Giommi A . Ear acupuncture in psychosomatic medicine: the importance of the Sanjiao (triple heater) area. Acupunct Electrother Res. (1993) 18:185–94. doi: 10.3727/036012993816357449, PMID: 7906478

[25] Li Y Hou L Wang Y Xie L Zhang M Pan Z . Auricular points acupressure for insulin resistance in overweight/obese women with polycystic ovary syndrome: protocol for a randomised controlled pilot trial. BMJ Open. (2019) 9:e027498. doi: 10.1136/bmjopen-2018-027498, PMID: 31142530 PMC6549699

[26] Zhou T Wang F Xu X Zhu Y Zhang R Lee HW . Non-pharmacological interventions of traditional Chinese medicine in treating polycystic ovary syndrome: a group consensus. Integr Med Res. (2024) 13:101093. doi: 10.1016/j.imr.2024.101093, PMID: 39967750 PMC11832912

[27] Li Y Zheng H Zheng Q Zhao L Qin E Wang Y . Use acupuncture to relieve perimenopausal syndrome: study protocol of a randomized controlled trial. Trials. (2014) 15:198. doi: 10.1186/1745-6215-15-198, PMID: 24886348 PMC4055374

[28] Liu LL Wan NJ Sun HH Zhang YM Lü YW . Effect of transcutaneous electrical acupoint stimulation combined with auricular acupressure on sexual hormone level and gonadal development in girls with precocious puberty. Zhen Ci Yan Jiu. (2023) 48:199–203. doi: 10.13702/j.1000-0607.20220011, PMID: 36858418

[29] Wang K Liu Z Xu B . Impact on the lipid level of obesity of spleen deficiency and damp blockage complicated by hyperlipemia treated with warm needling therapy and auricular acupuncture. Zhongguo Zhen Jiu. (2016) 36:225–30. 27344822

[30] Sun L Ma S Yu Y Li X Wei Q Min L . Transcutaneous auricular vagus nerve stimulation ameliorates adolescent depressive- and anxiety-like behaviors via hippocampus glycolysis and inflammation response. CNS Neurosci Ther. (2024) 30:e14614. doi: 10.1111/cns.14614, PMID: 38358062 PMC10867795

[31] Nielsen A Gereau S Tick H . Risks and safety of extended auricular therapy: A review of reviews and case reports of adverse events. Pain Med. (2020) 21:1276–93. doi: 10.1093/pm/pnz379, PMID: 32430505

[32] Xue K Wu H Jia L Yang S . Effectiveness and safety of auricular acupuncture for psoriasis: A protocol for systematic review and meta-analysis. Med (Balt). (2022) 101:e32020. doi: 10.1097/md.0000000000032020, PMID: 36401494 PMC9678494

[33] Lu M Sharmin S Tao Y Xia X Yang G Cong Y . Economic evaluation of acupuncture in treating patients with pain and mental health concerns: the results of the Alberta Complementary Health Integration Project. Front Public Health. (2024) 12:1362751. doi: 10.3389/fpubh.2024.1362751, PMID: 39386945 PMC11461202

[34] Cao HJ Li X Li XL Ward L Xie ZG Hu H . Factors influencing participant compliance in acupuncture trials: An in-depth interview study. PloS One. (2020) 15:e0231780. doi: 10.1371/journal.pone.0231780, PMID: 32298368 PMC7162473

[35] Wu T Liu Y Kong F Hu J Liu Y Yang J . Improvement of endocrine and metabolic conditions in patients with polycystic ovary syndrome through acupuncture and its combined therapies: a systematic review and meta-analysis. Ann Med. (2025) 57:2477295. doi: 10.1080/07853890.2025.2477295, PMID: 40091529 PMC11915742

[36] Wu Y Xiao Q Wang S Xu H Fang Y . Effectiveness of acupuncture for infertility in patients with polycystic ovary syndrome: A systematic review and network meta-analysis. Endocr Metab Immune Disord Drug Targets. (2024). doi: 10.2174/0118715303297819240826065755, PMID: 39313899 PMC13523171

[37] Ee C Smith CA Costello M Moran L Steiner GZ Stepto N . Acupuncture or auricular electro-acupuncture as adjuncts to lifestyle interventions for weight management in PCOS: protocol for a randomised controlled feasibility study. Pilot Feasibility Stud. (2020) 6:53. doi: 10.1186/s40814-020-00591-4, PMID: 32346487 PMC7183107

[38] Li YC Feng T He MJ . Effect of auricular point pressing bean method combined with metformin on insulin resistance in obese PCOS patients [in Chinese. Zhongguo Yixue Chuangxin. (2020) 17:153–7.

[39] Page MJ McKenzie JE Bossuyt PM Boutron I Hoffmann TC Mulrow CD . The PRISMA 2020 statement: an updated guideline for reporting systematic reviews. BMJ. (2021) 372:n71. doi: 10.1136/bmj.n71, PMID: 33782057 PMC8005924

[40] Geisthövel F . A comment on the European Society of Human Reproduction and Embryology/American Society for Reproductive Medicine consensus of the polycystic ovarian syndrome. Reprod BioMed Online. (2003) 7:602–5. doi: 10.1016/s1472-6483(10)62081-0, PMID: 14748954

[41] Higgins JPT Thomas J Chandler J Cumpston M Li T Page MJ eds. Cochrane handbook for systematic reviews of interventions (Version 6.0). Chichester (UK): The Cochrane Collaboration (2019).

[42] Dettori JR Norvell DC Chapman JR . Fixed-effect vs random-effects models for meta-analysis: 3 points to consider. Global Spine J. (2022) 12:1624–6. doi: 10.1177/21925682221110527, PMID: 35723546 PMC9393987

[43] Guyatt GH Oxman AD Vist GE Kunz R Falck-Ytter Y Alonso-Coello P . GRADE: an emerging consensus on rating quality of evidence and strength of recommendations. BMJ. (2008) 336:924–6. doi: 10.1136/bmj.39489.470347.AD, PMID: 18436948 PMC2335261

[44] Chen SY . Clinical study of auricular point pressing combined with Chinese medicine for obesity-type polycystic ovary syndrome (kidney deficiency and phlegm-dampness pattern). Shenyang: Liaoning University of Traditional Chinese Medicine (2021).

[45] Ling W . Clinical observation of Chinese medicine combined with auricular therapy for PCOS (phlegm-damp pattern). Changchun: Changchun University of Chinese Medicine (2015).

[46] Wan X Jiang L Yang YQ Feng HY Xu NN Li L . Clinical study of modified Cangfu Daotan Decoction combined with auricular therapy for phlegm-stasis type PCOS. Shaanxi Zhongyi. (2022) 43:1797–800.

[47] Zhang WF . Clinical observation on auricular acupoint pressure combined with modified Banxia Xiexin Decoction for gastric-heat and spleen-deficiency type PCOS-IR. Beijing: China Academy of Chinese Medical Sciences (2023).

[48] Zhu SQ . Clinical observation on Chai-Shao-Duonan-Yin combined with auricular acupoint therapy for kidney-deficiency and liver-depression type PCOS. Beijing: Beijing University of Chinese Medicine (2023).

[49] Zuo J . Clinical study of modified Fangfeng Tongsheng San combined with auricular pressing therapy for obesity-type polycystic ovary syndrome. Nanjing: Nanjing University of Chinese Medicine (2011).

[50] Gan L . Observation and nursing of auricular point pressing combined with metformin for polycystic ovary syndrome with obesity [in Chinese. Guangming Zhongyi. (2012) 27:2037–8.

[51] Li YC Feng T Rong CF He MJ . Effect of auricular intradermal needle combined with metformin on insulin resistance in phlegm-damp type PCOS patients [in Chinese. Zhongyi Waiti Zazhi. (2021) 30:59–61.

[52] Sun JY . Clinical observation on auricular point pressing combined with metformin in treating reproductive-age obesity-type PCOS-IR. Harbin: Heilongjiang University of Chinese Medicine (2023).

[53] Zhong L . Clinical observation on auricular point pressing combined with metformin in treating adolescent obesity-type PCOS-IR. Harbin: Heilongjiang University of Chinese Medicine (2023).

[54] Zhuang MD . Clinical observation on auricular point pressing combined with metformin in treating lipid metabolic disorder in adolescent obesity-type PCOS. Harbin: Heilongjiang University of Chinese Medicine (2023).

[55] Li LN Zhang YH Wang J . Effect of electroacupuncture combined with auricular point pressing on serum hormones and insulin levels in PCOS patients. Hunan Zhongyiyao Daxue Xuebao. (2015) 35:52–5.

[56] Li QQ Zhong WQ Zhang J Zeng RY Feng SL . Clinical observation of acupuncture combined with auricular point pressing in treatment of polycystic ovary syndrome. Shanghai Zhenjiu Zazhi. (2017) 36:895–9. doi: 10.13460/j.issn.1005-0957.2017.08.0895

[57] Liu HJ . Clinical study of electroacupuncture combined with auricular pressing in treating obesity-type PCOS based on body composition analysis. Nanjing: Nanjing University of Chinese Medicine (2016).

[58] Liu Y . Effect of electroacupuncture combined with auricular pressing on serum hormone and insulin levels in PCOS patients. Renren Jiankang. (2018) 22:102.

[59] Ma JJ . Effect of electroacupuncture combined with auricular pressing on polycystic ovary syndrome. Zhongguo Xiandai Yaowu Yingyong. (2017) 11:65–6. doi: 10.14164/j.cnki.cn11-5581/r.2017.21.034

[60] Zhang SK He H Wang Y Sun JJ Mo XL Liu J . Clinical observation on auricular vagus nerve stimulation in improving negative emotions in PCOS patients [in Chinese. Tianjin Zhongyiyao Daxue Xuebao. (2024) 43:30–6.

[61] Baskind NE Balen AH . Hypothalamic-pituitary, ovarian and adrenal contributions to polycystic ovary syndrome. Best Pract Res Clin Obstet Gynaecol. (2016) 37:80–97. doi: 10.1016/j.bpobgyn.2016.03.005, PMID: 27137106

[62] Walters KA Gilchrist RB Ledger WL Teede HJ Handelsman DJ Campbell RE . New perspectives on the pathogenesis of PCOS: neuroendocrine origins. Trends Endocrinol Metab. (2018) 29:841–52. doi: 10.1016/j.tem.2018.08.005, PMID: 30195991

[63] Wang J Wu D Guo H Li M . Hyperandrogenemia and insulin resistance: The chief culprit of polycystic ovary syndrome. Life Sci. (2019) 236:116940. doi: 10.1016/j.lfs.2019.116940, PMID: 31604107

[64] Hashim ZH Hamdan FB Al-Salihi AR . Autonomic dysfunction in women with polycystic ovary syndrome. Iran J Reprod Med. (2015) 13:27–34. 25653673 PMC4306982

[65] Saranya K Pal GK Habeebullah S Pal P . Assessment of cardiovascular autonomic function in patients with polycystic ovary syndrome. J Obstet Gynaecol Res. (2014) 40:192–9. doi: 10.1111/jog.12154, PMID: 24102794

[66] Li W Chen Y Xu L . Association of sympathetic nervous system activity with polycystic ovarian syndrome. Clin Exp Obstet Gynecol. (2014) 41:499–506. doi: 10.12891/ceog16592014 25864247

[67] Velusami D Sivasubramanian S . Sympathovagal imbalance and neurophysiologic cognitive assessment using evoked potentials in polycystic ovary syndrome in young adolescents - a cross-sectional study. J Basic Clin Physiol Pharmacol. (2018) 30:233–7. doi: 10.1515/jbcpp-2018-0081, PMID: 30332394

[68] Ondicova K Mravec B . Multilevel interactions between the sympathetic and parasympathetic nervous systems: a minireview. Endocr Regul. (2010) 44:69–75. doi: 10.4149/endo_2010_02_69, PMID: 20429636

[69] Shorakae S Abell SK Hiam DS Lambert EA Eikelis N Jona E . High-molecular-weight adiponectin is inversely associated with sympathetic activity in polycystic ovary syndrome. Fertil Steril. (2018) 109:532–9. doi: 10.1016/j.fertnstert.2017.11.020, PMID: 29428305

[70] Sverrisdóttir YB Mogren T Kataoka J Janson PO Stener-Victorin E . Is polycystic ovary syndrome associated with high sympathetic nerve activity and size at birth? Am J Physiol Endocrinol Metab. (2008) 294:E576–81. doi: 10.1152/ajpendo.00725.2007, PMID: 18198350

[71] Shorakae S Ranasinha S Abell S Lambert G Lambert E de Courten B . Inter-related effects of insulin resistance, hyperandrogenism, sympathetic dysfunction and chronic inflammation in PCOS. Clin Endocrinol (Oxf). (2018) 89:628–33. doi: 10.1111/cen.13808, PMID: 29992612

[72] Peuker ET Filler TJ . The nerve supply of the human auricle. Clin Anat. (2002) 15:35–7. doi: 10.1002/ca.1089, PMID: 11835542

[73] Trevizol A Barros MD Liquidato B Cordeiro Q Shiozawa P . Vagus nerve stimulation in neuropsychiatry: Targeting anatomy-based stimulation sites. Epilepsy Behav. (2015) 51:18. doi: 10.1016/j.yebeh.2015.07.009, PMID: 26262931

[74] He W Jing XH Zhu B Zhu XL Li L Bai WZ . The auriculo-vagal afferent pathway and its role in seizure suppression in rats. BMC Neurosci. (2013) 14:85. doi: 10.1186/1471-2202-14-85, PMID: 23927528 PMC3751281

[75] Zhu HH Rong PJ Chen Y Song XK Wang JY . Possible mechanisms of auricular acupoint stimulation in the treatment of migraine by activating auricular vagus nerve. Zhen Ci Yan Jiu. (2024) 49:403–8. doi: 10.13702/j.1000-0607.20221262, PMID: 38649209

[76] Usichenko T Hacker H Lotze M . Transcutaneous auricular vagal nerve stimulation (taVNS) might be a mechanism behind the analgesic effects of auricular acupuncture. Brain Stimul. (2017) 10:1042–4. doi: 10.1016/j.brs.2017.07.013, PMID: 28803834

[77] Morán C Franco A Morán JL Handal A Morales L Domínguez R . Neural activity between ovaries and the prevertebral celiac-superior mesenteric ganglia varies during the estrous cycle of the rat. Endocrine. (2005) 26:147–52. doi: 10.1385/endo:26:2:147, PMID: 15888926

[78] Morán C Zarate F Morán JL Handal A Domínguez R . Lateralization of the connections of the ovary to the celiac ganglia in juvenile rats. Reprod Biol Endocrinol. (2009) 7:50. doi: 10.1186/1477-7827-7-50, PMID: 19460167 PMC2697162

[79] Lawrence IE Jr. Burden HW Louis TM . Effect of abdominal vagotomy of the pregnant rat on LH and progesterone concentrations and fetal resorption. J Reprod Fertil. (1978) 53:131–6. doi: 10.1530/jrf.0.0530131, PMID: 641892

[80] Cruz ME Chávez R Domínguez R . Ovulation, follicular growth and ovarian reactivity to exogenous gonadotropins in adult rats with unilateral or bilateral section of the vagi nerves. Rev Invest Clin. (1986) 38:167–71. 3090668

[81] Waise TMZ Dranse HJ Lam TKT . The metabolic role of vagal afferent innervation. Nat Rev Gastroenterol Hepatol. (2018) 15:625–36. doi: 10.1038/s41575-018-0062-1, PMID: 30185916

[82] Teff KL . Visceral nerves: vagal and sympathetic innervation. JPEN J Parenter Enteral Nutr. (2008) 32:569–71. doi: 10.1177/0148607108321705, PMID: 18753395

[83] Bonaz B Sinniger V Pellissier S . Anti-inflammatory properties of the vagus nerve: potential therapeutic implications of vagus nerve stimulation. J Physiol. (2016) 594:5781–90. doi: 10.1113/jp271539, PMID: 27059884 PMC5063949

[84] Berthoud HR Powley TL . Characterization of vagal innervation to the rat celiac, suprarenal and mesenteric ganglia. J Auton Nerv Syst. (1993) 42:153–69. doi: 10.1016/0165-1838(93)90046-w, PMID: 8450174

[85] Wang H Yu M Ochani M Amella CA Tanovic M Susarla S . Nicotinic acetylcholine receptor alpha7 subunit is an essential regulator of inflammation. Nature. (2003) 421:384–8. doi: 10.1038/nature01339, PMID: 12508119

[86] Aboeldalyl S James C Seyam E Ibrahim EM Shawki HE Amer S . The role of chronic inflammation in polycystic ovarian syndrome-A systematic review and meta-analysis. Int J Mol Sci. (2021) 22. doi: 10.3390/ijms22052734, PMID: 33800490 PMC7962967

[87] Zhang Q Yang Z Ou X Zhang M Qin X Wu G . The role of immunity in insulin resistance in patients with polycystic ovary syndrome. Front Endocrinol (Laus). (2024) 15:1464561. doi: 10.3389/fendo.2024.1464561, PMID: 39911236 PMC11797073

[88] González F . Inflammation in Polycystic Ovary Syndrome: underpinning of insulin resistance and ovarian dysfunction. Steroids. (2012) 77:300–5. doi: 10.1016/j.steroids.2011.12.003, PMID: 22178787 PMC3309040

[89] Apovian CM Shah SN Wolfe BM Ikramuddin S Miller CJ Tweden KS . Two-year outcomes of vagal nerve blocking (vBloc) for the treatment of obesity in the reCharge trial. Obes Surg. (2017) 27:169–76. doi: 10.1007/s11695-016-2325-7, PMID: 27506803 PMC5187356

[90] Ikramuddin S Blackstone RP Brancatisano A Toouli J Shah SN Wolfe BM . Effect of reversible intermittent intra-abdominal vagal nerve blockade on morbid obesity: the ReCharge randomized clinical trial. Jama. (2014) 312:915–22. doi: 10.1001/jama.2014.10540, PMID: 25182100

[91] Val-Laillet D Biraben A Randuineau G Malbert CH . Chronic vagus nerve stimulation decreased weight gain, food consumption and sweet craving in adult obese minipigs. Appetite. (2010) 55:245–52. doi: 10.1016/j.appet.2010.06.008, PMID: 20600417

[92] Kozorosky EM Lee CH Lee JG Nunez Martinez V Padayachee LE Stauss HM . Transcutaneous auricular vagus nerve stimulation augments postprandial inhibition of ghrelin. Physiol Rep. (2022) 10:e15253. doi: 10.14814/phy2.15253, PMID: 35441808 PMC9020171

[93] Shen EY Hsieh CL Chang YH Lin JG . Observation of sympathomimetic effect of ear acupuncture stimulation for body weight reduction. Am J Chin Med. (2009) 37:1023–30. doi: 10.1142/s0192415x09007466, PMID: 19938213

[94] Li H Zhang JB Xu C Tang QQ Shen WX Zhou JZ . Effects and mechanisms of auricular vagus nerve stimulation on high-fat-diet--induced obese rats. Nutrition. (2015) 31:1416–22. doi: 10.1016/j.nut.2015.05.007, PMID: 26429664

[95] Liu CH Yang MH Zhang GZ Wang XX Li B Li M . Neural networks and the anti-inflammatory effect of transcutaneous auricular vagus nerve stimulation in depression. J Neuroinflamm. (2020) 17:54. doi: 10.1186/s12974-020-01732-5, PMID: 32050990 PMC7017619

[96] Kong J Fang J Park J Li S Rong P . Treating depression with transcutaneous auricular vagus nerve stimulation: state of the art and future perspectives. Front Psychiatry. (2018) 9:20. doi: 10.3389/fpsyt.2018.00020, PMID: 29459836 PMC5807379

[97] Yan L Li H Qian Y Zhang J Cong S Zhang X . Transcutaneous vagus nerve stimulation: a new strategy for Alzheimer’s disease intervention through the brain-gut-microbiota axis? Front Aging Neurosci. (2024) 16:1334887. doi: 10.3389/fnagi.2024.1334887, PMID: 38476661 PMC10927744

