# Supplementary material for: Effectiveness and safety of auricular therapy for polycystic ovary syndrome: a systematic review and meta-analysis
Source: Front Endocrinol (Lausanne). 2026 Mar 4;17:1726938. doi: 10.3389/fendo.2026.1726938 (PMC12995678; doi:10.3389/fendo.2026.1726938)
Supplement: Supplementary Material 2 — Full search strategy. [file Table2.docx]

**Supplementary Material 1. Complete Search Strategies**

The complete search strategies for each database are listed below:

**PubMed Search Strategy**
(ear[Title/Abstract] OR ear acupuncture[Title/Abstract] OR auricular therapy[Title/Abstract] OR auricular acupuncture[Title/Abstract] OR auricular acupressure[Title/Abstract] OR auricular acupoints[Title/Abstract] OR auricular point-sticking[Title/Abstract] OR auricular point pressing with bean[Title/Abstract])
AND
(polycystic ovary syndrome[Title/Abstract] OR polycystic ovarian syndrome[Title/Abstract] OR Stein-Leventhal syndrome[Title/Abstract] OR polycystic ovary disease[Title/Abstract] OR Syndrome, Polycystic Ovary[Title/Abstract] OR PCOS[Title/Abstract])

**EMBASE Search Strategy**
#1 'ear'/exp OR 'ear' OR 'ear acupuncture'/exp OR 'ear acupuncture' OR 'auricular therapy'/exp OR 'auricular therapy' OR 'auricular acupuncture'/exp OR 'auricular acupuncture' OR 'auricular acupressure'/exp OR 'auricular acupressure' OR 'auricular acupoints' OR 'auricular point-sticking' OR 'auricular point pressing with bean'
#2 'polycystic ovary syndrome'/exp OR 'polycystic ovary syndrome' OR 'polycystic ovarian syndrome' OR 'stein-leventhal syndrome'/exp OR 'stein-leventhal syndrome' OR 'polycystic ovary disease'/exp OR 'polycystic ovary disease' OR 'syndrome, polycystic ovary' OR 'PCOS'
#1 AND #2

**Web of Science Search Strategy**
(TS=(ear OR auricular point OR otopoint OR ear point OR auricular-plaster therapy OR ear point sticking OR auricular acupressure OR auricular point sticking))
AND
(TS=(polycystic ovary syndrome OR ovary syndrome, polycystic OR syndrome, polycystic ovary OR polycystic ovarian syndrome OR polycystic ovary syndrome OR PCOS OR PCO))

**The Cochrane Library Search Strategy**
(ear OR ear acupuncture OR auricular therapy OR auricular acupuncture OR auricular acupressure OR auricular acupoints OR auricular point-sticking OR auricular point pressing with bean):ti,ab,kw
AND
(polycystic ovary syndrome OR polycystic ovarian syndrome OR Stein-Leventhal syndrome OR polycystic ovary disease OR Syndrome, Polycystic Ovary OR PCOS):ti,ab,kw

**China National Knowledge Infrastructure (CNKI) Search Strategy**
("耳" + "耳针" + "耳穴" + "耳穴贴压" + "耳穴贴豆" + "耳穴压豆") * ("多囊卵巢综合征" + "多囊卵巢" + "PCOS")

**Wan Fang Database Search Strategy**
(耳针 OR 耳穴 OR 耳穴贴压 OR 耳穴贴豆 OR 耳穴压豆 OR 耳穴疗法)
AND
(多囊卵巢综合征 OR 多囊卵巢 OR PCOS)

**China Science and Technology Journal Database (VIP) Search Strategy**
("耳针" + "耳穴" + "耳穴贴压" + "耳穴贴豆" + "耳穴压豆" + "耳穴疗法" + "耳穴刺激") * ("多囊卵巢综合征" + "多囊卵巢" + "PCOS")

**Chinese Biomedical Literature Database (CBM) Search Strategy**
(("耳"[常用字段] OR "Ear"[常用字段] OR "前庭蜗神经器官"[常用字段] OR "前庭耳蜗系统"[常用字段] OR "耳"[主题词])
OR ("耳针"[常用字段] OR "耳针刺"[常用字段] OR "Ear Acupuncture"[常用字段] OR "耳廓针刺术"[常用字段] OR "耳廓针刺"[常用字段] OR "针刺, 耳"[主题词])
OR ("耳穴"[常用字段] OR "耳针穴位"[常用字段] OR "Auricular Acupuncture Points"[常用字段] OR "穴位, 耳针"[主题词])
OR ("耳穴疗法"[常用字段] OR "Auriculotherapy"[常用字段] OR "耳针疗法"[常用字段] OR "耳穴疗法"[主题词])
OR "耳穴刺激"[常用字段]
OR ("耳穴贴压"[常用字段] OR "耳穴贴压疗法"[常用字段] OR "Auricular Plastertherapy"[常用字段] OR "耳穴埋豆疗法"[常用字段] OR "耳穴埋籽"[常用字段] OR "耳穴埋针"[常用字段] OR "耳穴贴压疗法"[主题词])
OR "耳穴贴豆"[常用字段] OR "耳穴压豆"[常用字段])
AND
(("多囊卵巢综合征"[常用字段] OR "Polycystic Ovary Syndrome"[常用字段] OR "Stein-Leventhal综合征"[常用字段] OR "Sclerocystic卵巢变性"[常用字段] OR "Sclerocystic卵巢综合征"[常用字段] OR "硬化囊状性卵巢"[常用字段] OR "Sclerocystic的卵巢"[常用字段] OR "多囊卵巢综合征"[主题词])
OR "多囊卵巢"[常用字段] OR "PCOS"[常用字段]))
