# Supplementary material for: Effectiveness and safety of auricular therapy for polycystic ovary syndrome: a systematic review and meta-analysis
Source: Front Endocrinol (Lausanne). 2026 Mar 4;17:1726938. doi: 10.3389/fendo.2026.1726938 (PMC12995678; doi:10.3389/fendo.2026.1726938)
Supplement: Supplementary Figure 4 — Figure S1. [file Table4.docx]

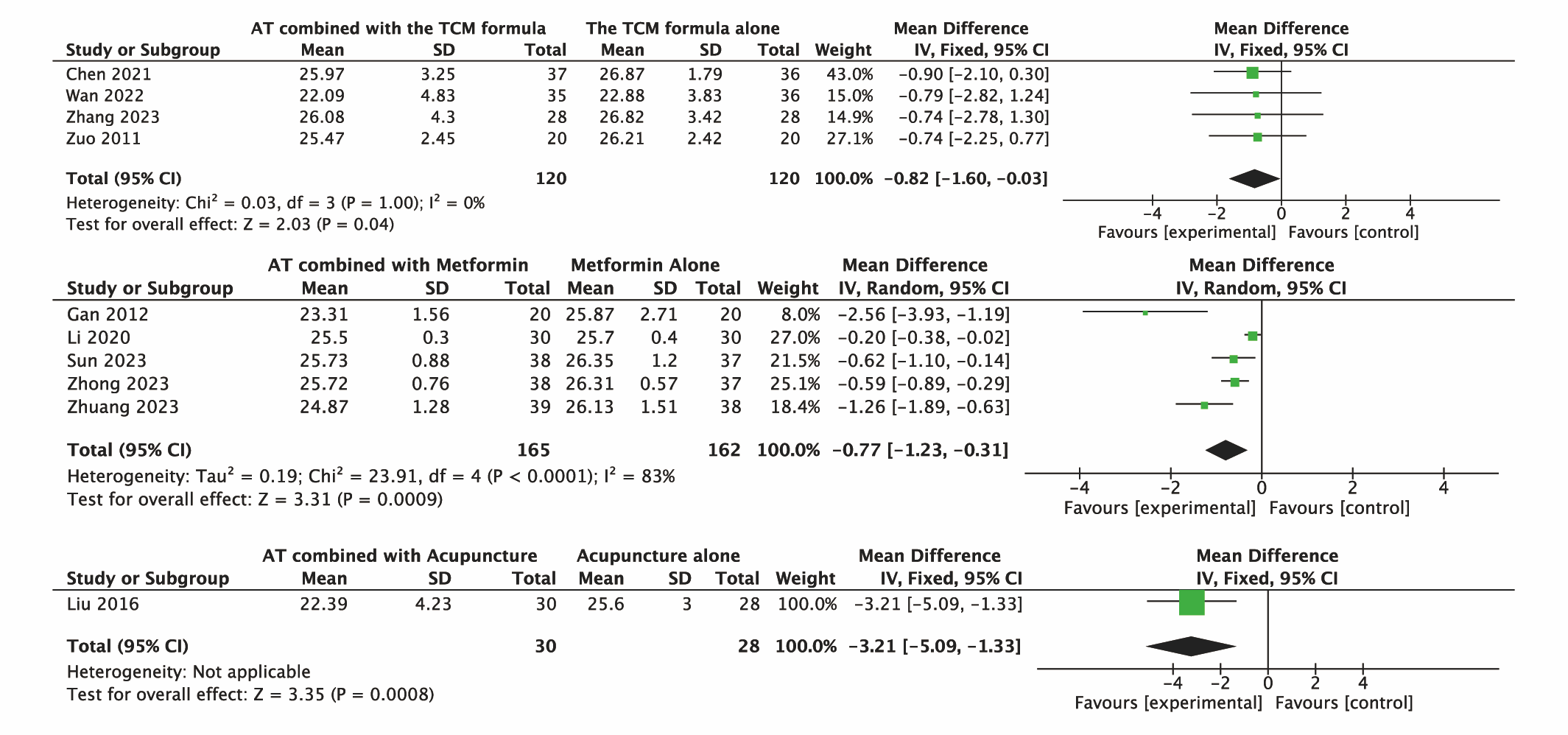

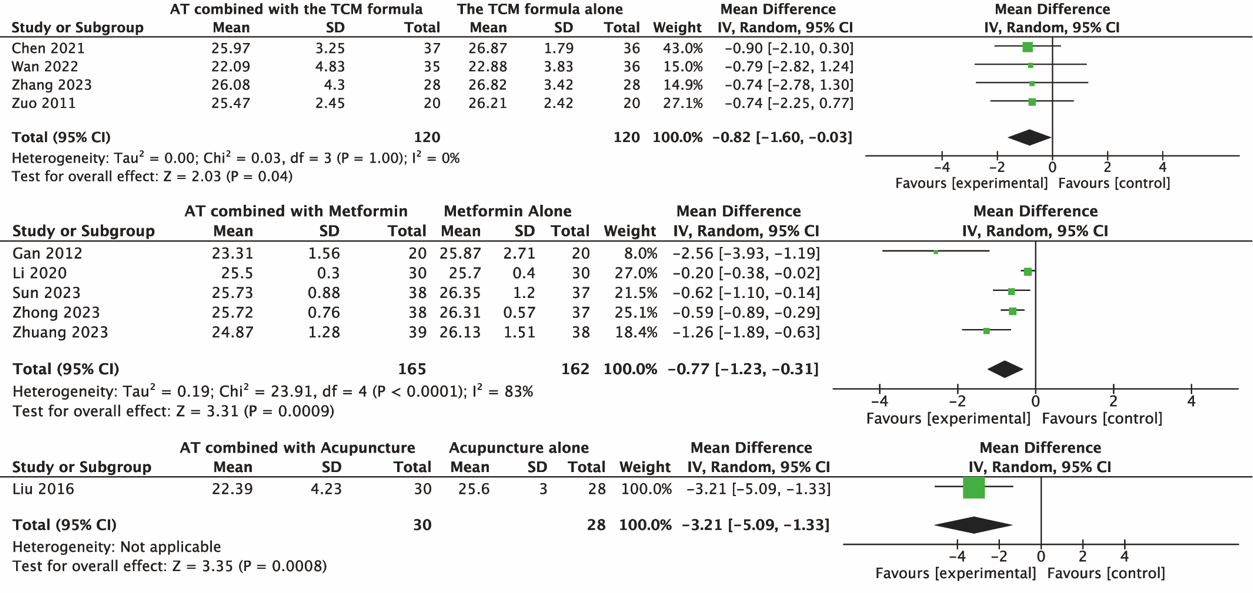
**Figure S4 legends**

Sensitivity analyses using both fixed-effect and random-effects models yielded consistent estimates for BMI, indicating that the pooled effect was robust to the choice of meta-analytic model despite differences in between-study heterogeneity across subgroups.
